# Non-linear Mendelian randomization: evaluation of effect modification in the residual and doubly-ranked methods with simulated and empirical examples

**DOI:** 10.1007/s10654-025-01208-x

**Published:** 2025-06-02

**Authors:** Fergus W. Hamilton, David A. Hughes, Tianyuan Lu, Zoltán Kutalik, Apostolos Gkatzionis, Kate Tilling, Fernando P. Hartwig, George Davey Smith

**Affiliations:** 1 MRC Integrative Epidemiology Unit, University of Bristol, Bristol, UK; 2 Infection Science, North Bristol NHS Trust, Bristol, UK; 3 Pennington Biomedical Research Center, Baton Rouge, LA USA; 4 Lady Davis Institute for Medical Research, Montreal, QC Canada; 5 Department of Statistical Sciences, University of Toronto, Toronto, Canada; 6 Department of Population Health Sciences, University of Wisconsin-Madison, Madison, WI USA; 7 Department of Biostatistics and Medical Informatics, University of Wisconsin-Madison, Madison, WI USA; 8 Department of Computational Biology, University of Lausanne, Lausanne, Switzerland; 9 Swiss Institute of Bioinformatics, Lausanne, Switzerland; 10 University Center for Primary Care and Public Health, Lausanne, Switzerland; 11 Postgraduate Program in Epidemiology, Federal University of Pelotas, Pelotas, Brazil

**Keywords:** Mendelian randomisation, NLMR, Methods, Biostatistics

## Abstract

Non-linear Mendelian randomisation (NLMR) is a relatively recently developed approach to estimate the causal effect of an exposure on an outcome where this is expected to be non-linear. Two commonly used techniques—based on stratifying the exposure and performing Mendelian randomisation (MR) within each strata—are the residual and doubly-ranked methods. The residual method is known to be biased in the presence of genetic effect heterogeneity—where the effect of the genotype on the exposure varies between individuals. The doubly-ranked method is considered to be less sensitive to genetic effect heterogeneity. In this paper, we simulate genetic effect heterogeneity and confounding of the exposure and outcome and identify that both methods are susceptible to likely unpredictable bias in this setting. Using UK Biobank, we identify empirical evidence of genetic effect heterogeneity and show via simulated outcomes that this leads to biased MR estimates within strata, whilst conventional MR across the full sample remains unbiased. We suggest that these biases are highly likely to be present in other empirical NLMR analyses using these methods and urge caution in current usage. Simulated outcome analyses may represent a useful test to identify if genetic effect heterogeneity is likely to bias NLMR estimates in future analyses.

**Supplementary Information:**

The online version contains supplementary material available at 10.1007/s10654-025-01208-x.

## Introduction

Mendelian randomisation (MR) is an established approach in epidemiology and cognate disciplines, using the principles of random allocation at conception, consequent on Mendel’s Laws, to strengthen causal inference regarding the effect of a potential exposure on an outcome [1–3]. Extensions to this technique allow for estimation of non-linear effects of the exposure on the outcome. Two such methods, based upon stratification of the exposure, have been widely used. In the ‘residual’ method, developed in 2014 [4], the exposure is regressed onto the genotype, and the residuals from this regression are used to generate strata. In each stratum, MR is performed and these MR estimates used to generate a dose–response curve [5]. This method has strong assumptions of homogeneity in the genotype-exposure relationship—referred to as the ‘constant genetic effect’ assumption [6]. The residual method has been widely used to identify non-linearities in exposure-outcome relationships [7–13]. However, some of the results generated by this method in practice [8, 10, 11] are not possible given assumptions of the method and the known distribution of data [14, 15]. Indeed, the authors of one retracted NLMR vitamin D paper [8] commented that what they had reported was a “logical impossibility” [16] Given these issues—which also raise concern about the validity of the results of other papers applying the residual method—a novel approach was introduced, the doubly-ranked method [17]. In the doubly-ranked method, strata are generated in multiple steps. In the first step, the population is ranked by the level of the genetic instrument into pre-strata. Subsequently, within each pre-stratum, the participant with the lowest level of the exposure is taken and placed into the lowest stratum. The participant with the next lowest level of the exposure in pre-stratum 1 is placed into stratum 2. The same process occurs for all pre-strata. The first stratum therefore contains the first participant of every pre-stratum, each of which has the lowest level of exposure in the pre-stratum (Supplementary Figure 1). A more detailed explanation is given in the paper introducing the method [17].

The doubly-ranked method does not rely on the constant genetic effect assumption to generate unbiased estimates. Instead, a weaker assumption—the “rank-preserving assumption”—requires that an individual’s exposure ranking would be the same for all values of an instrument. If this assumption holds, then the doubly-ranked method is valid even in the presence of non-linearity or heterogeneity in the genotype-exposure association [17].

However, there has been little reported research into the conditions in which genetic effect heterogeneity leads to violation of the rank-preserving assumption, and how often such conditions arise in practice. The paper introducing the doubly-ranked method conducted a small-scale simulation study in which the method appeared to be robust to genetic effect heterogeneity; motivated by that, the authors concluded:

the doubly-ranked method can obtain unbiased stratum-specific estimates and appropriate coverage rates even when the effect of the instrument on the exposure is non-linear or heterogeneous [17].

While it is possible for the doubly-ranked method to obtain unbiased causal effect estimates in the presence of heterogeneity, it is not clear how likely that is in real-data analyses. In this paper, we aimed to (a) assess whether genetic effect heterogeneity biases estimates from the doubly-ranked and residual methods (we define bias below), and (b) provide evidence on whether genetic effect heterogeneity is likely to bias estimates in empirical analyses.

## Materials and methods

### Simulation studies

We report our simulations in line with the “Aims, data-generating mechanisms, estimands, methods, and performance measures” (ADEMP) framework [18]. We include example R scripts to describe our simulation approach and allow replication in our GitHub repository (https://github.com/gushamilton/simulated_nlmr/).

### Aims

Our aim was to assess the impact of genetic effect heterogeneity on overall and stratum-specific estimates from non-linear MR using the doubly-ranked and residual methods.

### Data generating process

Data were generated in R, using the *faux* package [19]. Each simulation included the following basic setup, where we simulated variables for* g*, representing our instrumental variable, $u$ and $v$ representing other variables, and errors for *x* and* y* ($e_{x}$ and $e_{y}$). We then generated our exposure (*x*) and outcome (*y*) using the formulae below, with different values for each of the terms depending on the simulation, although we fixed the effect of *g* on *x* to be 0.3 for all simulations. Where $b_{gux}$ is not 0, there is genetic effect heterogeneity.

1 $$\begin{aligned} & x = 0.3g + b_{ux} u + b_{gux} gu + b_{vx} v + e_{x} \\ & y = b_{uy} u + b_{vy} v + e_{y} \\ \end{aligned}$$

Variables were simulated using the *rnorm_multi* function in *faux*, with a mean of 2 and an SD of 1 for all variables. In each simulated cohort, we included 100,000 simulated participants. We then performed one-sample MR in this simulated cohort.

Linear MR was performed using the Wald ratio across the whole dataset, after calculating the IV-exposure effect and IV-outcome effect [1]. For non-linear MR, we stratified the data using the doubly-ranked or residual method then used the Wald ratio estimator within each stratum. We chose to use ten strata for all analyses, similar effects were shown using other strata numbers (data not shown). Except where stated, all analyses were performed unadjusted for covariates.

### Performance measures and estimands

In all of our simulations, there was no causal effect of the exposure on the outcome. Therefore, we know the expected value for MR estimates across the whole cohort and within each stratum should be 0. We also know there should be no strata-specific heterogeneity in the MR effect. We therefore considered two definitions of bias; a) firstly if any stratum specific estimate deviated from 0, and b) if there was heterogeneity across strata in the MR effect.

To assess bias in stratum specific estimates from the doubly ranked and residual methods, we calculated the average (across strata) of the square of each stratum-specific estimate, and then averaged these across 100 replicates of each simulation (mean squared error, MSE). Boxplots were used to visualise the distribution of replicate stratum-specific estimates.

To identify heterogeneity in estimates across strata, we calculated the p-value for Cochran’s Q across strata within each replicate using the *meta* package in R. To summarise this, we then calculated the proportion of replicates in which there was nominal evidence of heterogeneity (p < 0.05). If this value was 1, then all 100 replicates had nominal evidence of heterogeneity, while if this value was 0, no replicate had any evidence of heterogeneity.

### Violations of the rank-preserving assumption

To simulate the violations of the rank preserving assumption; we used a similar simulation set up as above, but used 50,000 participants, and 100 replicates. We varied input parameters to generate effect heterogeneity as described in the results text and in our R code. Code to perform the simulation is available at our GitHub page. All model settings (e.g. distributions of variables) are described in the text or in our R code.

### Empirical analyses

All empirical analyses in this study were performed in UK Biobank. This study recruited around 500,000 participants between 2006 and 2010 from 22 sites across the UK [20]. Participants were invited via post and had a range of interviews on recruitment to record previous life events, demographics, and medical history. Additionally, participants had blood samples taken for biochemical testing and had a range of physical and anthropometric measures performed (e.g. body mass index, blood pressure). Subsequently, participants had record linkage to electronic health record data for secondary care and national death data (for > 99% of participants).

We analysed data on the subset of participants who were minimally related and of European ancestry (n = 385,290), defined as per [21]. Specifically, we used the MRC Integrative Epidemiology Unit genetically quality controlled data, described in detail elsewhere [21], excluding highly related participants.

### Exposures and data source

This analysis focused on six exposures (Body Mass Index [BMI], serum 25-hydroxy vitamin D [Vitamin D], and four lipid traits: high density lipoprotein cholesterol [HDL], low density lipoprotein cholesterol [LDL], triglycerides [TG], and lipoprotein(a) [Lp(a)]. BMI was measured in participants on recruitment to UK Biobank. Details on measurement are available with the companion paper [20] and via the UK Biobank website. For other exposures (Vitamin D and lipid traits), these were measured in blood. We used the sample taken on recruitment where participants had multiple samples. Our study population for each analysis included only participants who had these values recorded and had genetic data available.

To identify variants associated with BMI to use as an instrument, we used a large recent study which meta-analysed available summary statistics but did not include UK Biobank [22]. We extracted all variants which were associated with BMI (p < 5 × 10^–8^) and then clumped them by linkage disequilibrium to include only independent variants (r^2^ < 0.001, kb = 10,000). LD clumping was performed using the *TwoSampleMR* package in R. In total we included 68 variants (Supplementary Table S1). To generate variants associated with Vitamin D and HDL, LDL and TG, we used the same approach as described in our recent paper (Supplementary Tables S2, S3) [23]. Finally, for Lp(a), we chose only *cis* variants from a recent analysis of lipoprotein A [7]. Included variants for Lp(a) are reported in Supplementary Table S4. To generate an individual PRS for each participant we used PLINK v2.0.4 [24], with the summary effect generated by weighted allele scores, with each allele weighted by its effect on the exposure.

We simulated our ‘confounded’ exposures and outcomes, from the observed variables as below.

2 $$\begin{aligned} & g = measured\;PRS \\ & x = measured\;exposure + b_{ux} u \\ & y = b_{uy} u + e_{y} \\ \end{aligned}$$

we simulate $u$ and $e_{y}$, using the same approach as above (normally distributed variables, with a mean of 2 and SD of 1). We generated our ‘confounded’ exposures and outcome using the formulae above, on scaled exposures to aid comparison between exposures. We visualized any bias in stratum specific estimates using the same plotting approach as in our simulation studies. We did not calculate MSE as there was clear visual evidence of bias and it would be challenging to compare across exposures.

### Ethics

This study was performed under the UK Biobank application number 81499. UK Biobank was ethically approved by the North West Multi-centre Research Ethics Committee (MREC).

### Data and code availability

This analysis consists of both simulated work and empirical work. For the simulated work, code is available to replicate key findings in our GitHub (https://github.com/gushamilton/simulated_nlmr). We are unable to share data from our empirical analyses due to data privacy concerns in UK Biobank, but these data can be obtained by bona fide researchers.

## Results

### Stratum-specific estimates are biased in the presence of IV-exposure effect modification and unmeasured exposure-outcome confounding

**Fig. 1:**
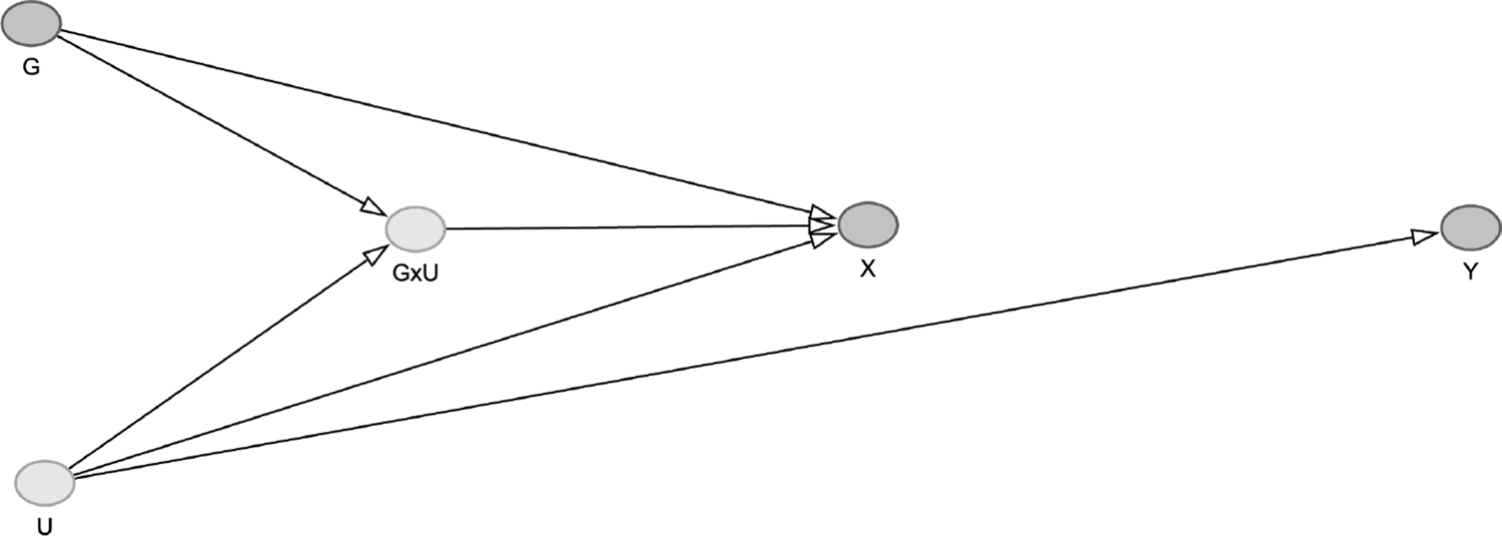
DAG of Simulation A This shows an effect of an IV (G) on X and Y in the presence of an unmeasured confounder U which interacts with the effect of G onto X. We show the interaction as a node in the DAG in line with recent literature [25, 26].

We first explored a simple simulation of genetic effect heterogeneity, whereby the effect of the genotype on the exposure is modified by another variable. To do this, we generated a simulated sample of 100,000 participants, with a single, instrumental variable ($$g$$), a continuous exposure ($$x$$), a continuous outcome ($$y)$$, and a confounder ($$u$$) with the following settings (Simulation A, Fig. 1):

3 $$\begin{aligned} & x = 0.3g + b_{ux} u + b_{gux} gu + e_{x} \\ & y = b_{uy} u + e_{y} \\ \end{aligned}$$

There was no causal effect of the exposure on the outcome, and the association between the exposure and the outcome was completely due to the confounder *u*. Our interest lies in whether an interaction effect ($b_{gux} \ne 0)$ would lead to bias in MR estimates, the size of this bias, and whether the strength of the confounding effects $b_{ux}$ and $b_{uy}$ affected the magnitude of bias.

We first explored the impact of the g-by-u interaction effect size by fixing $b_{ux}$ = 0.3 and $b_{uy}$ = 0.3 and varying $b_{gux}$ ∈ {− 0.1, − 0.02, 0, 0.02, 0.1}. When $b_{gux}$ = 0, there was no g-by-u interaction, and thus no genetic effect heterogeneity. In a second set of simulations, we explored the impact of the confounding effect size by fixing $b_{gux}$ = − 0.1, and separately varying $b_{ux} \in${0.3, 0.05, 0} and $b_{uy} \in${0.3, 0.05, 0}. The IV-exposure effect $b_{x}$ = 0.3 was held constant for all simulations. Each simulation scenario was replicated 100 times and the results were averaged across replications.

**Fig. 2:**
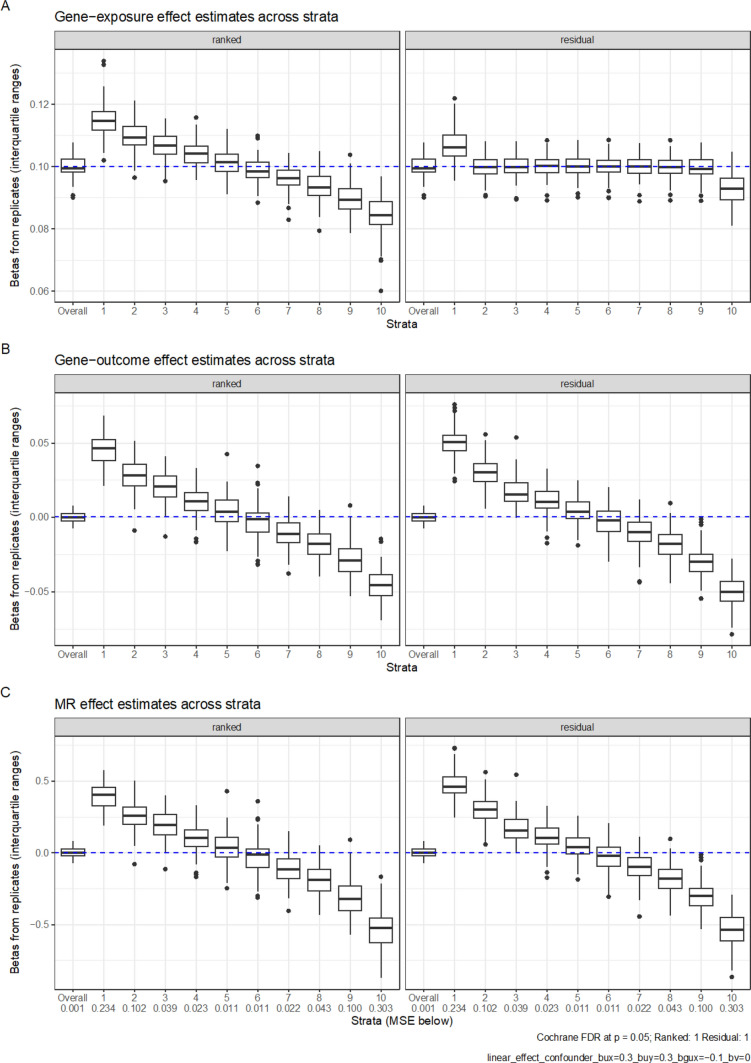
Simulation results for X = 0.3G + 0.3U − 0.1GU + e_x_ and Y = 0.3U + e_Y_, Boxplots represent the estimates from each replicate. In total 100 replicates were performed, with each sample containing 100,000 people. Plot A shows IV-exposure estimates across 10 strata; Plot B shows IV-outcome estimates across 10 strata; Plot C shows MR estimates across 10 strata. The FDR is the proportion of replications that meet a nominal significance for heterogeneity using Cochran’s Q for estimates across strata. The MSE represents the mean squared error in each strata. The top and bottom of the box represent the 25th and 75th centile, while whiskers represent the point closest to 1.5 × the IQR below and above the box. Points outside that are plotted individually

In the presence of a confounding effect ($b_{ux}$ = $b_{uy}$ = 0.3) and a strong g-by-u interaction effect ($b_{gux}$ = − 0.1), both the residual and doubly-ranked method obtained positive IV-outcome associations and MR estimates in the lower strata, as well as negative IV-outcome associations and MR estimates in the higher strata (Fig. 2), suggesting bias. These spurious associations attenuated with a weaker g-by-u interaction effect ($b_{gux}$ = − 0.02; Supplementary Figure 2). Importantly the confidence interval for the conventional, full-sample MR estimate overlapped with the null. When there was no g-by-u interaction ($b_{gux}$ = 0), there was no longer bias in any stratum (Supplementary Figure 3). This trend was consistent when the g-by-u interaction had the opposite effect direction (Supplementary Figures 4 and 5), although the strata specific estimates were in the other direction (negative estimates in lower strata, and positive estimates in upper strata).

**Fig. 3:**
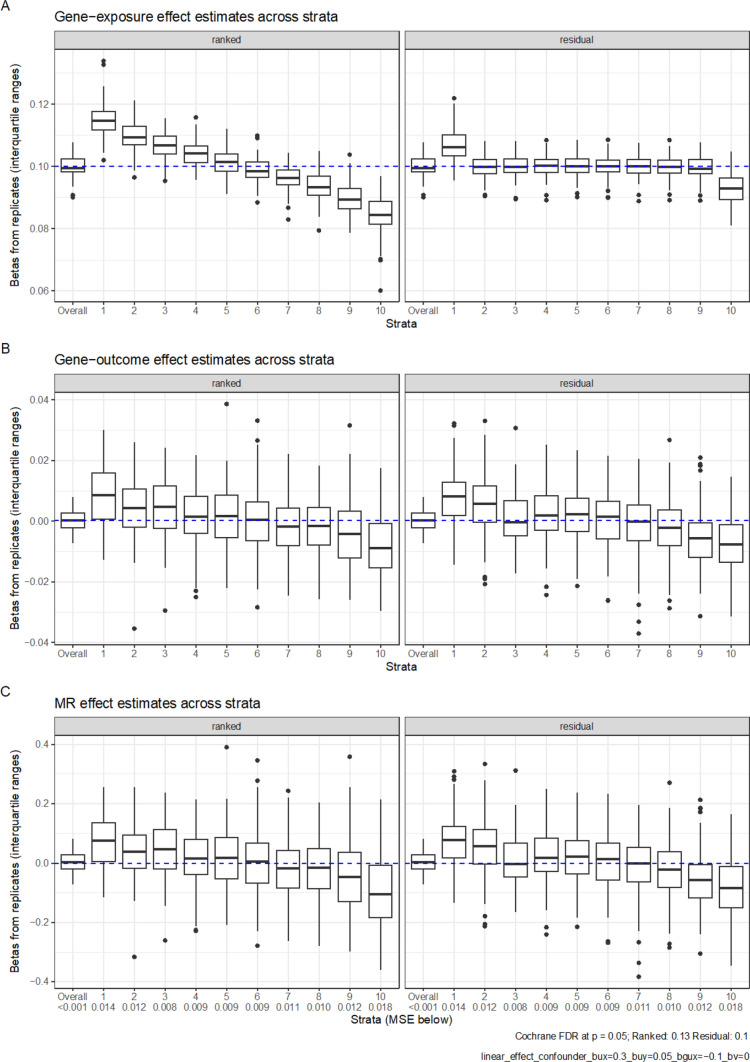
Simulation results of a linear exposure-outcome association X = 0.3G + 0.3U − 0.1GU + e_x_ and Y = 0.05U + e_Y_. Boxplots represent the estimates from each replicate. In total 100 replicates were performed, with each sample containing 100,000 people. Plot A shows IV-exposure estimates across 10 strata; Plot B shows IV-outcome estimates across 10 strata; Plot C shows MR estimates across 10 strata. The FDR is the proportion of replications that meet a nominal significance for heterogeneity using Cochran’s Q for estimates across strata. The MSE represents the mean squared error in each strata. The top and bottom of the box represent the 25th and 75th centile, while whiskers represent the point closest to 1.5 × the IQR below and above the box. Points outside that are plotted individually

The magnitude of the effect of the confounder on the outcome ($b_{uy}$) had a substantial influence in both methods. When this confounding effect lessened ($b_{uy}$ = 0.05), bias in per-stratum MR estimates decreased accordingly (Fig. 3). Adjusting for the confounder also recovered unbiased estimates in all simulation scenarios. However, when incomplete adjustment occurred (by generating a variable *u*_*error*_ that was not perfectly correlated with *u* (*u*_*error*_*,* Pearson’s R 0.8 with *u*) and using this as would occur when *u* was measured with error), bias was still present (Supplementary Figure 7).

**Fig. 4:**
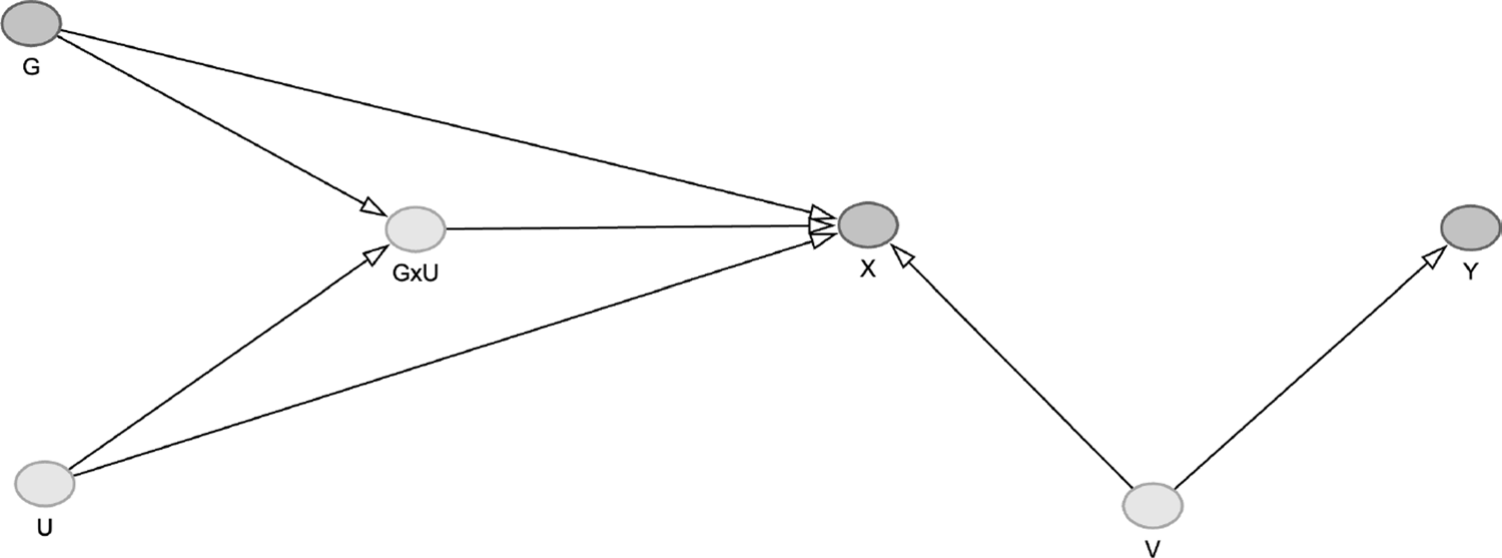
Simulation study B: we now include another variable V, that confounds the relationship between the exposure and outcome, independent of the interaction between G and U

We then ran a second simulation where there are two independent unmeasured factors: (i) *u*, which modifies the IV-exposure effect ($b_{{{\text{gux}}}}$ = − 0.1), but does not confound the exposure and outcom ($b_{uy}$ = 0); and (ii) *v*, which does not modify the IV-exposure effect, but is a confounder of the exposure and outcome. (Simulation B, Fig. 4).

4 $$\begin{aligned} & x = 0.3g + b_{ux} u + b_{gux} gu + b_{v} v + e_{x} \\ & y = b_{v} v + e_{y} \\ \end{aligned}$$

**Fig. 5:**
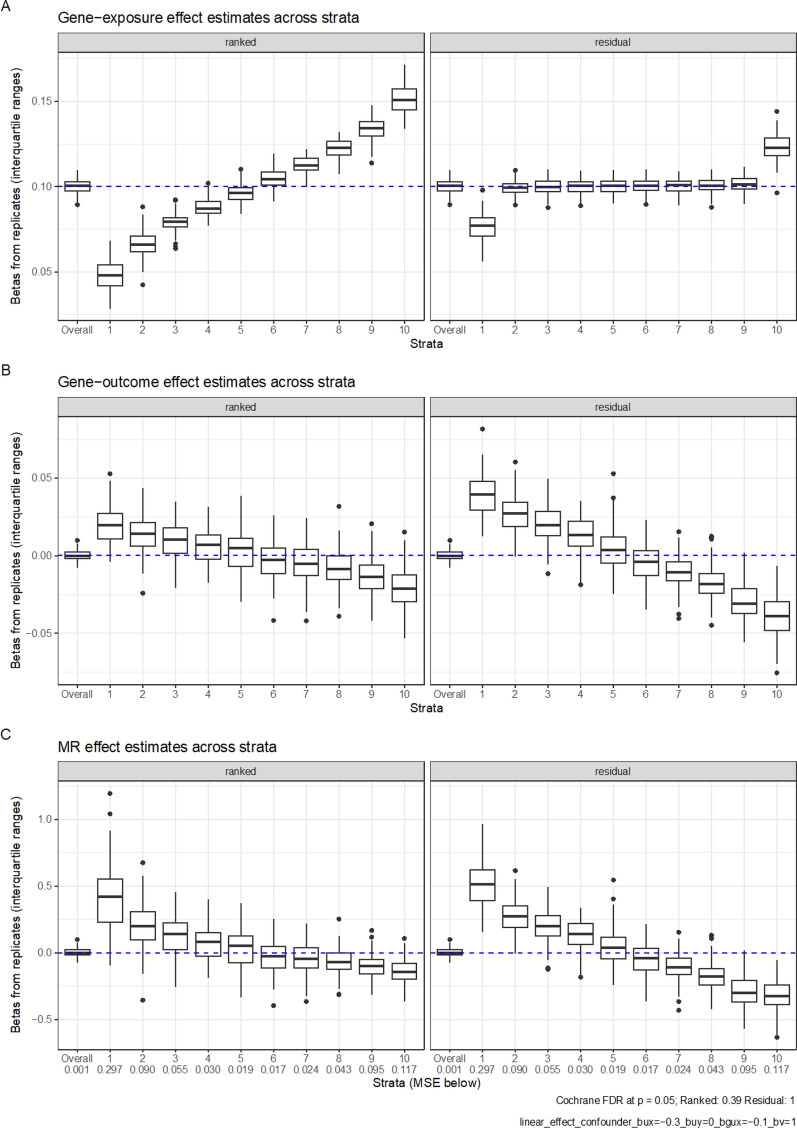
Simulation results of a linear exposure-outcome association X = 0.3G − 0.3U + − 0.1GU + V + e_x_ and Y = 1 V + e_Y_. Boxplots represent the estimates from each replicate. In total 100 replicates were performed, with each sample containing 100,000 people. Plot A shows IV-exposure estimates across 10 strata; Plot B shows IV-outcome estimates across 10 strata; Plot C shows MR estimates across 10 strata. The FDR is the proportion of replications that meet a nominal significance for heterogeneity using Cochran’s Q for estimates across strata. The MSE represents the mean squared error in each strata. The top and bottom of the box represent the 25th and 75th centile, while whiskers represent the point closest to 1.5× the IQR below and above the box. Points outside that are plotted individually

**Fig. 6:**
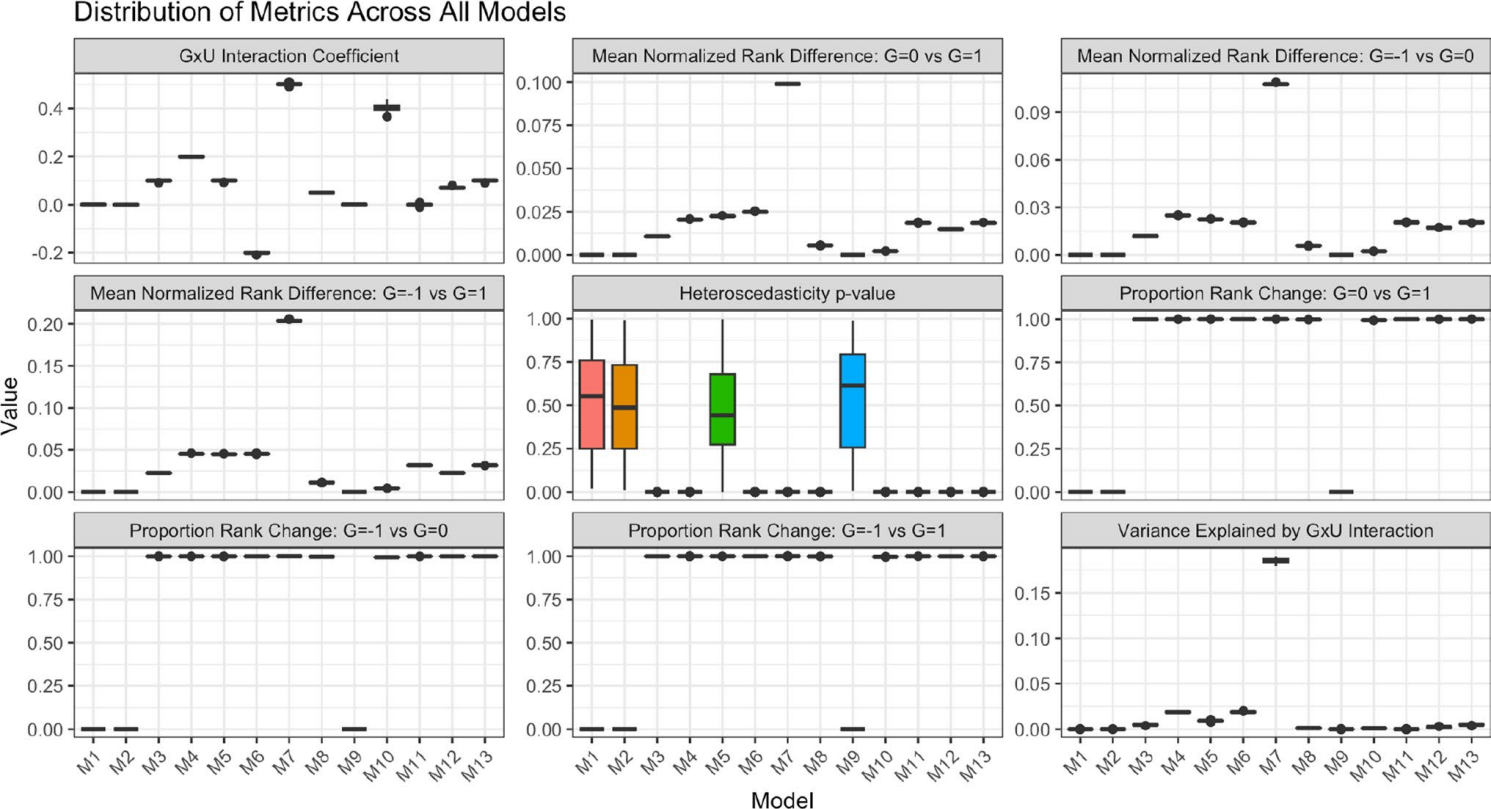
Simulation testing the rank-preserving assumption. Each panel represents a different metric of the model, while each column represents a different model. The set up for all tested models is shown in Table 1. Mean normalised rank-difference refers to the absolute mean difference in ranks divided by the total number of participants. For example, if there were ten people, and the absolute average rank difference of each participant when G = 1 to when G = 0 was 3, this would be a value of 0.3. The proportion of rank change simply represents the number of participants who had a different rank for a different value of G. Boxplots are presented from 100 simulations

When $b_{ux}$ = − 0.3 was fixed and $b_{v} \in${0.3, 1} varies we observed a bias in both gene-outcome and MR effect estimates (Fig. 6, $b_{v}$ = 1; Supplementary Figure 8, $b_{v}$ = 0.3). Figure 5 MR estimates were biased and were positive in lower strata and negative in higher strata. When $b_{ux}$ = 0.3 bias remained but with a reversal of direction (i.e. negative MR estimates in lower strata, positive MR estimates in upper strata). When $b_{ux}$ = 0 but $b_{gux}$ remained = − 0.1, bias was present, with positive MR estimates in lower strata and lower MR estimates in upper strata, although the magnitude of bias was reduced compared to when $b_{ux} \ne 0$.

In sum, these analyses suggested that it is sufficient for bias to exist when there is both:

- The presence of a variable that modifies the effect of the genotype on the exposure.
  *And*
- Unmeasured exposure-outcome confounding.

Importantly, the variable(s) that interact with the IV in its effect on the exposure do not need to be the same variables that confound the exposure outcome relationship. The direction and magnitude of bias is related to the (a) direction and size of the interaction ($$b_{gux}$$), (b) the direction and size of the confounder-outcome ($$b_{ux}$$), (c) the presence, direction and size of confounder-outcome ($$b_{uy} )$$ effect, and (d) the direction and size of an exclusive exposure-outcome ($$b_{vy}$$ and $$b_{vx}$$) confounder. It is important to note that under certain (likely rare) conditions NLMR estimates can be unbiased in this situation; which is where the confounding variable is perfectly uncorrelated with *x* in the population. This is an unlikely situation which we discuss further later, but occurs when simulating an IV and confounder with means of 0.

### Comparisons with previous simulations suggest the degree of bias relates to breaking of the rank preserving assumption

It seems likely that these biased estimates in the doubly-ranked method are generated by violation of the rank-preserving assumption. We then replicated prior work to compare with our simulations. We chose 9 models from our simulations above, added one further quadratic model for the genotype-exposure relationship, and also tested four models of the genotype-exposure effect from the two previous papers. These are listed in Table 1.

**Table 1:** Descriptions of models tested for violation of the rank-preserving assumption

Model	Description	Equation
M1	Linear model with G and U	$X = 0.3G + U + \varepsilon$
M2	Linear model with reduced U effect	$X = 0.3G + 0.1U + \varepsilon$
M3	Interaction model with small GxU effect	$X = 0.3G + U + 0.1GU + \varepsilon$
M4	Interaction model with large GxU effect	$X = 0.3G + U + 0.2GU + \varepsilon$
M5	Interaction model without U effect	$X = 0.3G + 0.1GU + \varepsilon$
M6	Interaction model with negative GxU effect	$X = 0.3G + U - 0.2GU + \varepsilon$
M7	Interaction model with large GU effect but small U effect	$X = 0.3G + 0.1U + 0.5GU + \varepsilon$
M8	Interaction model with very small GxU effect	$X = 0.3G + U + 0.05GU + \varepsilon$
M9	Quadratic G model	$X = 0.3G + 0.1G\wedge 2 + U + \varepsilon$
M10	Tian et al., *PLOS Genetics* model^a^	$X = - 10 + \left( {1.5 + 0.4U} \right)\left( {G + 5} \right) + U + \varepsilon$
M11	Burgess *Human Heredity* model (genetic effect depends on X)	$X = \alpha G + U + \varepsilon ,$ $where\;\alpha \sim N\left( {0.3 + 0.1\varepsilon , 0.1\wedge 2} \right)$
M12	Burgess *Human Heredity model (*genetic effect depends on *X and U equally)*	$X = \alpha G + U + \varepsilon ,$ $where \;\alpha \sim N\left( {0.3 + 0.1/\surd 2\left( {\varepsilon + U} \right), 0.1\wedge 2} \right)$
M13	Burgess *Human Heredity* model (genetic effect depends on U only)	$X = \alpha G + U + \varepsilon ,$ $where\;\alpha \sim N\left( {0.3 + 0.1U, 0.1\wedge 2} \right)$

^a^Note the SD of g here was fitted to be 0.5^2^ to replicate their approach

All used normally distributed variables for the exposure, outcome, and any other variables. We simulated 50,000 observations for each model. For our simulations, we set the mean value for *g*, *u* and all error terms to be 0, with an SD of 1 for all simulations except the *PLOS Genetics* (Model M10, see Table 1) analysis where the SD for g was 0.5^2^, with the other standard deviations being 1, in line with their analysis. We can therefore directly compare across the models.

We then fixed *g* at 3 values (− 1 SD below the mean, mean, and + 1SD above the mean), and using the same model and with the already simulated values for u, computed the value of the exposure for each value of *g*, and ranked each participant by the exposure. If the rank-preserving assumption holds, a participant’s rank should be unaffected by this change in genotype level and therefore the ranks should be equal. For each simulation we calculated (a) the number of participants that had the exact same rank when *g* = − 1SD;* g* = 0 and *g* =  + 1SD, (b) the absolute mean difference in rank for a participant when *g* = − 1SD, *g* = 0, and *g* =  + 1SD, (c) and model metrics including the proportion of variance explained by any interaction and the GxU interaction coefficient. Simulations were run 100 times. Results are shown in Fig. 6

These results confirm that the rank-preserving assumption was violated in the simulations when there is a GxU interaction. When there was an interaction, the degree of violation appeared to relate to the size of the interaction effect and the variance explained by the interaction. These results also help explain the apparent discordance between the results of the previous simulations on effect heterogeneity and our results. In the 2023 *Human Heredity* paper [6], it is shown that the rank preserving assumption is broken and there are therefore differences between levels of confounders across strata. These are consistent with our simulations. In the *PLOS Genetics* [17] paper, we also see violation of the rank preserving assumption, although this is weaker than in our simulations, with a lower mean normalized rank difference. MR estimates in the doubly-ranked method in the original paper are reported as close to the null, but closer inspection of them identifies that they are (weakly) biased, and so are consistent with our simulations and the *Human Heredity* paper. The degree of bias is more visible when the sample size is increased so there is more precision around estimates: we replicate their analysis using a sample size of 100,000 and 350,000 (representing most analyses in e.g. UK Biobank) in Supplementary Figure 9 and show that the estimates are more clearly biased when a larger number of participants are included. As a final note, we note that Model 5, which simulates GxU interaction but no effect of *u* on *x* (except through the interaction) violates the rank preserving assumption but would not lead to biased estimates. That is because in this (hypothetical and empirically unlikely) situation, *u* and *x* are uncorrelated despite the GxU interaction. Therefore, in this single situation there is violation of the rank preserving assumption but no bias.

### The rank-preserving assumption is not violated for non-linear functions of g-x

Finally, previous simulations have not shown biased estimates from the doubly-ranked method when there is a non-linear relationship between *g* and *x*. In Model 10, we model a non-linear function of the genotype-exposure relationship, and find that it does not break the rank-preserving assumption. We then proved this to be true (see Supplementary Note 1 for proof) for any given non-linear function of *g* given additive effects of g and u, and a constant dose–response curve. Therefore, non-linear genotype exposure relationships do not break the rank-preserving assumption. This is an important strength of the doubly-ranked method when compared to the residual approach, since the constant genetic effect assumption is violated when the effect of the IV on the exposure is non-linear.

### Summary of simulations

In these simulations, we considerably extend the previous work by Small [27] and the authors of the residual and doubly-ranked method [4–6, 17]. We show that biased estimates can occur in the presence of unmeasured confounding when it occurs when there is genetic effect heterogeneity whether that is via interaction with another variable (which does not have to be an exposure-outcome confounder). Given unmeasured confounding is likely in nearly all settings where MR is likely to be used (it is indeed the major reason for applying MR [2, 28]), this means that the requirement for bias in practical terms is the presence of any effect modification of the IV-exposure relationship. Finally, we reconcile our results with previous simulations and identify that methods that lead to biased MR estimates violate the rank-preserving assumption that underlies the doubly-ranked method. We therefore moved on to explore whether such interactions occur in an empirical setting and whether the size of the interaction effects we simulate are relevant, as this appears to be the critical question.

### Variables that interact with the IV-exposure relationship exist in real data

One plausible variable that interacts with the genotype and the exposure is ‘ill-health’. That is, for many exposures, the effect of the genotype on the exposure may vary depending on a person’s general health status. If this interaction occurs, all that is required for bias is additional unmeasured confounding of the exposure-outcome relationship. Additionally, as ill-health is challenging to define, it would not be possible to reliably measure and adjust for it, as even if it were possible to define it, measurement error would compromise the adjustment. As such, if ill-health interacts with an IV-exposure relationship, biased NLMR estimates are likely in the presence of exposure-outcome unmeasured confounding.

We therefore tested for interactions between a proxy for ill-health and genetic variants used to instrument 6 common exposures: BMI, Vitamin D, HDL cholesterol, LDL cholesterol, triglycerides and lipoprotein A. We defined ill-health in two separate ways. Firstly, we identified those who had above the 80th quantile for levels of C-reactive protein, Cystatin-C (a marker of renal function), and below the 20th quantile for albumin, forced expiratory flow volume in 1 s (FEV_1_), and those who had a history of dementia, liver disease, or cancer prior to recruitment to UK Biobank. Each of these was worth a single point, so the most ill participants had a potential score of 7 (although the actual maximum recorded score was 6), while the healthiest individuals had a score of 0. This we called the summary illness score. Secondly, to provide a more granular definition of ill health, we ranked each participant on the above 4 quantitative variables, so, for example, the participant with the highest CRP had the highest rank, while those with the lowest FEV_1_ had the highest rank. Each participant has then 4 separate ranks (for each quantitative variable). We then calculated the median of these ranks for each participant to generate their overall ill health ranking.

We scaled this variable to have an SD of one and mean of 0 to aid interpretability. We confirmed that both of these scores strongly associated with early mortality in UK Biobank (defined as death before 11th November 2021, our cut off period, Supplementary Figure 10), although with differing effects: those with a summary illness score of 0 had a mortality 3.8% (n = 138,229), while the 15 participants with a score of 6 had a mortality of 53%. In contrast we saw a weaker relationship of early mortality with the median ranking: those in decile 1 had a mortality of 2.8% (n = 38,529), while those in decile 10 had a mortality of 16.5% (n = 38,529).

We then tested for a statistical interaction between each IV and both illness scores in their effect on the exposure (i.e., whether the association between the IV and the exposure differs between levels of the illness scores). For every IV-exposure relationship we tested, except Lp(a), and for both illness measures, we found strong statistical evidence of an interaction. The size of the interaction effects we identified was in many cases comparable to the interaction effects we simulated above (e.g. the interaction beta for IV_vitD_:Illness rank was − 0.20; for IV_[vitD]_:BMI 0.14 standard deviations) (Table 2).

**Table 2:** Interaction effects of both illness definitions for 6 commonly used exposures

Sickness rank	Estimate (SE)	*P* value	Illness summary score	Estimate (SE)	*P* value
*BMI*					
IV_BMI_:Illness rank	0.141 (0.007)	2 × 10^–88^	IV_BMI_:Illness score	0.162 (0.008)	2 × 10^–86^
*Vitamin D*					
IV_vitD_:Illness rank	− 0.2 (0.037)	5 × 10^–8^	IV_vitD_:Illness score	− 0.16 (0.039)	4 × 10^–5^
*LDL*					
IV_LDL_:Illness rank	− 0.007 (0.001)	1 × 10^–6^	IV_LDL_:Illness score	− 0.004 (0.002)	0.02
*TG*					
IV_TG_:Illness rank	0.044 (0.002)	8 × 10 ^−155^	IV_TG_:Illness score	0.011 (0.002)	5 × 10 ^−9^
*HDL*					
IV_HDL_:Illness rank	− 0.007 (0.001)	2 × 10^–26^	IV_HDL_:Illness score	− 0.003 (0.001)	8 × 10^–6^
*Lp(a)*					
IV_Lp(a):Illness_ rank	0.074 (0.089)	0.41	IV_Lp(a):Illness_ score	0.093 (0.095)	0.32

Each PRS is scaled to have an SD of 1 and mean of 0. Estimates are on the effect of one standard deviation of the exposure

**Fig. 7:**
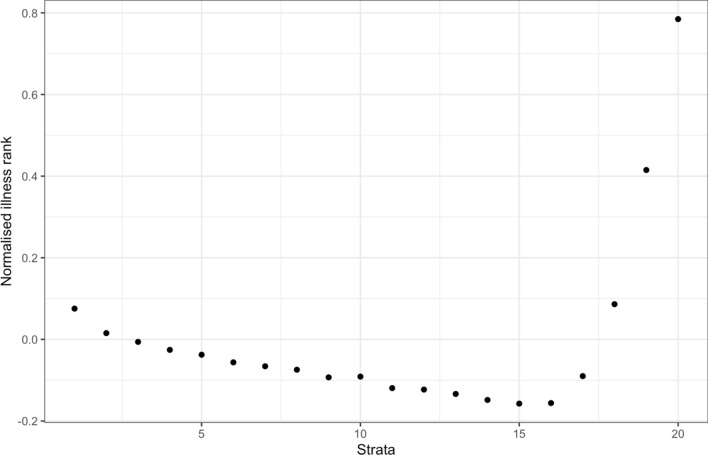
Median illness rank (scaled to have a mean of 0 and SD of 1) across doubly-ranked strata of Vitamin D. It can be seen that the least ‘ill’ population are those with moderately high vitamin D, but those with extremely high Vitamin D are the most ill, but the relationship is reverse J-shaped

To visualise one potential interaction, we plotted the level of the sickness score against strata generated by the doubly-ranked method for Vitamin D (Fig. 7). This shows that levels of ill-health are not linearly associated with Vitamin D strata, with ill health at both extreme ends of Vitamin D strata (despite a broadly linear marginal association between Vitamin D and sickness).

To demonstrate that ill-health is just one of many potential variables that could potentially lead to heterogenous genetic effects, we then analysed whether a marker of deprivation, the Townsend Deprivation Index [29] showed evidence of interaction with the genotype (Supplementary Table 5). For all exposures except LDL there was at least nominal evidence (p < 0.05) of an interaction effect. Importantly, despite levels of Lp(a) being largely genetically determined (75–90% heritable [30]) and so potentially less susceptible to gene-environment interaction, we still identified evidence of an interaction between Townsend Deprivation Index and the polygenic risk score for Lp(a) (beta − 0.165; p = 4 × 10^–9^). This was in the absence of a marginal association between Townsend Deprivation Index and Lp(a) (beta –0.048, p = 7.26 × 10^–2^). These data suggested that—at least for these commonly used exposures, interactions with the environment that which could potentially bias NLMR estimates were present and of a similar size to that in which we simulated (and in some cases larger).

### Simulating exposure-outcome relationships can uncover bias generated by genotype-exposure relationships in empirical analyses

**Fig. 8:**
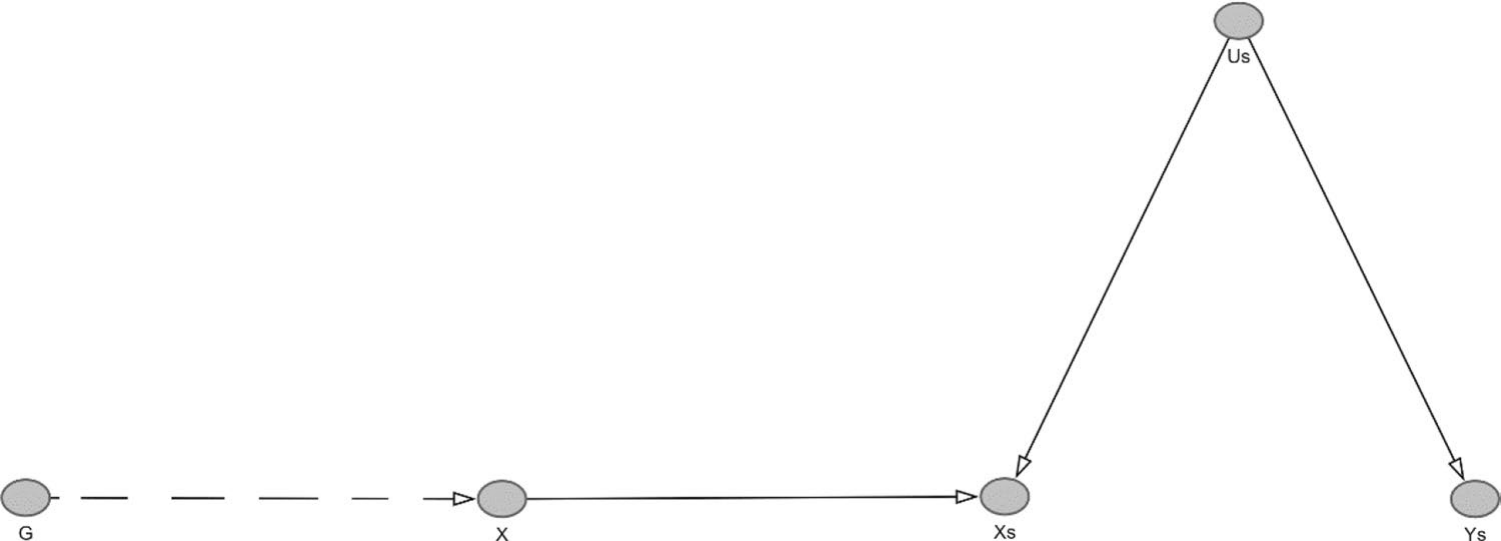
DAG of the simulated exposure outcome relationship. In this DAG, X is the true exposure, which is downstream of G. The dashed lines indicate we do not know the function that relates G to X. U_S_ is the simulated confounder, while X_S_ is the simulated exposure which is downstream of both the true exposure and unmeasured confounding. Y_s_ is the simulated outcome, and only relates to the confounder U_s_

We then went on to explore whether we could uncover bias that was likely to be generated by genetic effect heterogeneity. One way to do this is by using simulated outcomes. In this situation, we take a real exposure (e.g. BMI), and then use this to generate a simulated exposure (*x*). We separately simulate an outcome. We can then generate confounding of the simulated exposure-simulated outcome relationship. This was done by adding an unmeasured confounding variable *u* (Fig. 8).

7 $$\begin{aligned} & x = BMI + b_{ux} u \\ & y = b_{uy} u + e_{y} \\ \end{aligned}$$

This approach aims to simulate the bias that which could be encountered in an analysis where: (i) the instrument satisfies the three core IV assumptions (see Supplementary Note 2 for a proof of why the core IV assumptions are satisfied in these analysis); (ii) there is unmeasured confounding of the exposure and outcome; (iii) there is no causal effect of simulated exposure on simulated outcome on anyone in the studied population. Since the core IV assumptions hold, any bias can only be due to violations of some additional assumptions that a method requires. In the case of the methods under consideration, such additional assumptions are: (a) constant causal dose-response curve - i.e., the function describing the effect of the exposure on the outcome is the same for all individuals in the studied population; and (b) constant genetic effect (residual method) or rank preservation assumption (doubly-ranked method). Given point (iii), the constant causal dose-response curve assumption is satisfied. Therefore, violations of the assumptions under (b) are the only source of bias in these analyses.

We then ran NLMR on this confounded simulated exposure—simulated outcome relationship, by fixing $b_{ux}$ = − 1 and $b_{uy}$ = 1. We tested the same six commonly used exposures in this analysis, with details of the SNPs included in each IV in the supplementary tables. Each exposure was scaled to have an SD of 1 and a mean of 0 prior to this analysis so effect sizes can be compared across exposures. We performed 100 replicates as in our earlier simulations (further details in Methods).

**Fig. 9:**
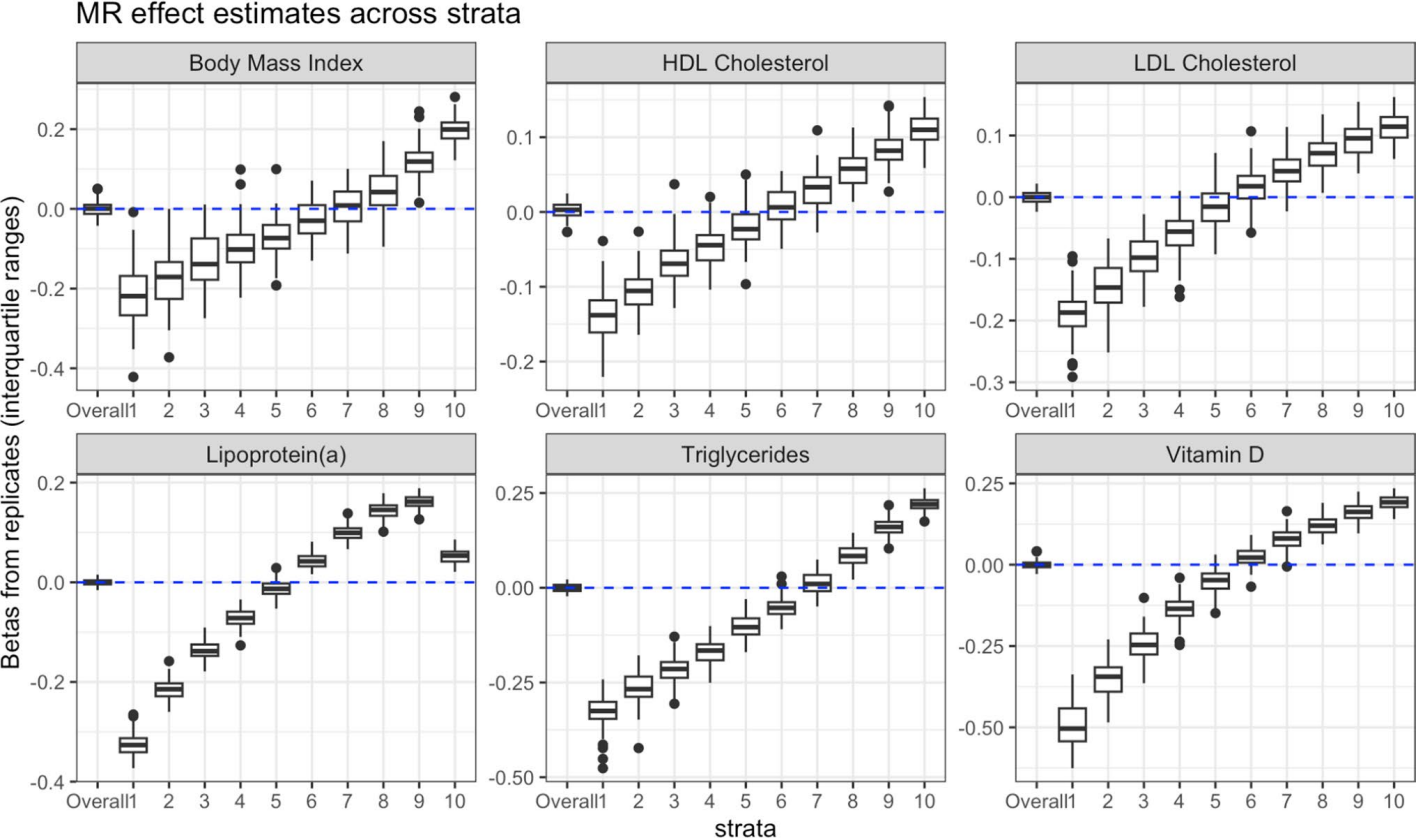
MR estimates from the confounded empirical analyses. Estimates are from the doubly-ranked method. Stratum specific estimates are on the Y-axis, with the overall effect in the first column, then individual strata specific estimates in each subsequent row

In this analysis, conventional MR estimates were null, as expected. However, NLMR estimates were biased for all exposures, with strongly negative estimates in lower strata, and strongly positive estimates in upper strata in both the residual and doubly-ranked method (Fig. 12 for the doubly ranked method and Supplementary Figure 11 for residual method). Interestingly, for Lp(a), but for no other exposure, we identified that estimates in strata 10 (i.e. the top strata) were much closer to the null. The size of this bias differed depending on the exposure, with the largest strata specific bias observed for Vitamin D. Although bias was generally greater in the residual method, in some situations, there was greater bias in the doubly-ranked method (e.g. for Vitamin D) (Fig. 9).

 As explained above, because the exposure and outcome data are simulated, possible violations of one or more of the core IV assumptions (for example, due to the strong selection that exists in the UK Biobank, or some other mechanism) cannot explain the bias in the estimates, since the design of the simulations ensure the core IV assumptions are all satisfied. Moreover, the simulation set-up guarantees that the causal dose-response curve is the same for all individuals. Therefore, these biases can only be explained by violations of either the constant genetic effect assumption (for the residual method) or the doubly-ranked assumption (for the doubly-ranked method). Such violations could be influenced by selection - e.g., it is possible that they only exist due to selection - as explained in Supplementary Note 2.

In an exploratory analysis, we went on to examine how individual SNPs were distributed across the strata when stratifying real exposures using both methods, and whether individual SNPs themselves had heteroskedastic effects. We then went on to test whether this bias could be reduced by limiting our IVs to SNPs that were not heteroskedastic. We first focussed on BMI as an exposure, and tried to identify if we could dissect the effect of individual SNPs that comprised our IV (Supplement 1). We identified that the vast majority of SNPs that make up our IV are heteroskedastic. Whether this is due to interactions or true variance effects is unknown. In the residual method, it is clear that these SNPs are not randomly distributed across strata, with weaker evidence for the doubly-ranked method. Importantly, we identified that by removing SNPs that had the strongest evidence of heteroskedasticity, the evidence of heterogeneous strata-specific IV-exposure effects (“non-constant genetic effects”) was greatly reduced.

## Discussion

Non-linear Mendelian randomization has become a commonly used and influential approach in genetic epidemiology. Recent data have challenged whether estimates from either the residual or doubly-ranked method should be considered reliable—with some studies being self-refuting [8, 11, 15], whilst others are clearly producing seriously biased findings [23], whilst not reaching the high bar of being literally impossible [14]. Published simulation studies are limited in scope and unclear on whether bias is present in the situation of genetic effect modification in the doubly-ranked method [6, 17]. In our paper, we identified that genetic effect heterogeneity can lead to biased NLMR estimates in both methods, and resolve the apparent contradictions in previous simulations on the role of genetic effect heterogeneity in generating bias, which can be attributed to the extent of violation of the rank-preserving assumption. It is worth noting that these issues of genetic effect heterogeneity were identified in a commentary by Small [27] on the original exposition of the residual method [4] and we expand on his work.

The primary result of our simulation study is that all that is required for bias in both methods is genetic effect heterogeneity and confounding of the exposure and outcome. As confounding of the exposure and the outcome can be assumed for nearly all situations in which MR is considered (if there was no confounding, then MR would have limited value), then all that is required for bias is the presence of any GxE or GxG interaction. For many empirical situations this seems likely, with several papers identifying interaction effects [25, 33–35]—and we highlight Nagpal and Gibson’s recent work identifying a large number of PRS by exposure interactions that generally reveal increased genetic effects in those with poorer overall health [35].

The question is therefore not whether this bias is present in empirical analyses, it is about the magnitude of this bias; which relates—in our simulations—to the size of the interaction effect. To provide evidence that this was likely to be an issue in reality we searched for a plausible variable that would have a large effect on many exposures, interact with many IV-exposure effects (on the additive scale) and be difficult to define and measure (making adjustment for it partial at best). We chose ill-health and used two definitions for this. For all exposures we tested except Lp(a), which is strongly genetically determined [30], we identified large interaction effects (of a similar scale to those used in our simulations). We then tested for a similar interaction with a marker of deprivation, and identified a different pattern of effects, with evidence of an interaction with Lp(a) in this case. Of course, these represent just some of many plausible interacting variables, and we do not make the claim that these factors will be relevant in all empirical analyses, simply that interaction effects of the magnitude required to bias NLMR results are likely and have been identified for some common exposures.

We then went on to simulate the effect of confounding using the real IV-exposure relationship for the same six widely used exposures. In essence, this is just an extension of our prior simulations, with the confounding generated in the same way. The degree of bias in these simulations was large and should lead to concern about the validity of any estimates in empirical analyses, at least when analysing these exposures using the UK Biobank. Of note, this analysis combining the real (IV-exposure relationship) and simulated (exposure and outcome) data could be performed at least as a falsification test in future empirical NLMR studies. We would recommend that authors of NLMR papers present such simulated data to show the degree of bias that might be expected from genetic effect heterogeneity, by performing similar analyses. Code for a falsification test is presented in our GitHub which will output a plot that can provide evidence of the potential for bias due to non-homogenous genetic effects.

We advocate again [23] for further methodological work being produced. In particular, further evaluation of when our above partially simulated analyses can predict bias in empirical analyses would be valuable. We suggest the research community should remain sceptical of estimates generated by the methods at present as genetic effect heterogeneity is likely present in real situations, and the direction and size of bias appears unpredictable. Finally, we tested whether removal of SNPs that were heteroskedastic might reduce this bias. We include this to suggest that this may be one potential approach that could be worth exploring further, rather than recommending this as a definitive sensitivity analysis. Researchers should also be aware that there are other approaches to generating dose–response curves using genetics, which are less likely to be biased. For example, it is possible to obtain some evidence on the shape of the relationship between an exposure and outcome through the use of subgroups of the population that have large differences in exposure levels and can be defined by characteristics that are unlikely colliders—such as sex or geographical region of residence. Such an approach has demonstrated that alcohol has adverse consequences on overall CVD at all levels [36]. It is also becoming increasingly possible, with expanding exome and whole genome sequencing data available in large studies, to construct allelic series which stretch much further across the range of an exposure and allow direct estimation of non-linear effects.

Ongoing use of the residual method should be carefully considered. It can generate implausible findings and appears to simply recapitulate the observational associations, as it does in the retracted or corrected Vitamin D [8] and BMI cases [12, 40]. Unsurprisingly when applied to HDL cholesterol the method simply recapitulates [13], but assigns causality to, the observational findings of strong protection, despite this being erroneous [37], exactly as in the cases of vitamin D and BMI in smokers. Our results highlight an important strength of the doubly-ranked method. Unlike the residual method, it is robust (in the absence of heterogeneity) even if the IV-exposure association is non-linear. This indicates that not all forms of heteroscedasticity of the exposure according to the IV would bias this method. For example, if the IV-exposure model is mis-specified (i.e. the relationship is modelled as linear, but it is acutally log-linear), then the residuals will be heteroscedastic with respect to the IV even in the absence of effect heterogeneity. This will not by itself lead to bias. Future methodological work may identify less stringent (and therefore more plausible) versions of the rank-preserving assumption that allow for valid causal inference.

Our findings have implications regarding empirically assessing the plausibility of the constant genetic effect and rank-preserving assumptions. As mentioned above, our analysis combining real and simulated data could be applied as a falsification test. Further work is also needed to determine the utility of variance tests to identify whether IVs that exhibit heteroskedasticity are invalid IVs for NLMR. One important weakness of this approach would be the risk of falsely rejecting the rank preserving assumption by mis specifying the IV-exposure model, as described above.

In summary, combined with previous work [23], this work suggests considerable caution should be applied if researchers feel the need to use either the residual or doubly-ranked approach. Both methods seem highly susceptible to bias in the generation of stratum-specific estimates under conditions that are likely present in practice, currently are not accompanied by empirical tools for the reliable detection of bias, and such biases are unpredictable in direction and magnitude. A method which essentially incorporates the residual approach for facilitating stratification in MR studies [38] should also undergo further testing before it is utilized. Triangulation [39] of results in all cases will be critical for interpretation of findings.

### Limitations

This paper presents a limited simulation of effect modification in NLMR with additional empirical analysis. One important consideration regarding our simulations is that they did not include scenarios where there is a non-zero causal effect. Additionally, the inputs to similation studies can clearly influence the results and conslusions drawn, sp from them. We therefore attempted to choose realistic values for our effects, and these were similar to empirical data from UK Biobank. In general, we would generally expect the performance of methods in the real world to be worse than in perfect simulations, given the additional known challenges with e.g. pleiotropy, population stratification, selection biases and difficulty to correctly model the dose–response curve of the effect of the exposure on the outcome.

Our analysis of real data relies entirely on UK Biobank, which is recognised to be subject to selection bias that can bias linear and non-linear MR estimates [40]. However, as our supplementary note clarifies, selection bias should not be a primary issue in our analyses. This result proves that, in this analysis, any selection bias (or any other factor) affecting the relationship between the genotype and exposure in UKB cannot result in the PRS being an invalid IV for the effect of the simulated exposure and simulated outcome.

Our pure simulation studies are also—by nature—free of these biases. Moreover, even if some of the biases we identify in the empirical analysis are specific to the UK Biobank, it should be noted that this is the most used dataset for NLMR, which implies that our findings may apply to most of the published studies using this method. Finally, in the few studies which use NLMR methods in UK Biobank and other studies potentially less biased by participation, negligible the findings from the different cohorts have been identified. For example, the predicted non-linear causal effects on BMI on mortality between UK Biobank and HUNT (a Norwegian study with a much higher response rate, and hence less potential for selection bias) are highly similar in both the residual [12] and ranked methods [40]. Similarly, there is little evidence of a major difference in predicted causal effects of Vitamin D on mortality in UK Biobank as compared to two Copenhagen based cohort studies that are much less subject to participation bias [8, 16]. Therefore, there is little evidence that selection bias is a major factor biasing NLMR results or that the biases are in any way specific to UK Biobank.

## Conclusion

Two commonly used non-linear MR methods can produce seriously biased estimates in the presence of genetic effect heterogeneity, with unpredictable directions and size of effects. In particular, the presence of genetic effect heterogeneity and unmeasured confounding of the exposure and outcome is all that is required for bias. Researchers should be highly sceptical of estimates generated by either method [41], and if a decision is made to use one of these methods, we advocate that use of the doubly-ranked method is favoured over the residual method.

## Supplementary Information

Below is the link to the electronic supplementary material.

Supplementary file1 (DOCX 692 kb)

Supplementary file2 (DOCX 1176 kb)

Supplementary file3 (XLSX 52 kb)

Supplementary file4 (DOCX 24 kb)
