# Supplementary material for: Non-linear Mendelian randomization: evaluation of effect modification in the residual and doubly-ranked methods with simulated and empirical examples
Source: Eur J Epidemiol. 2025 Jun 2;40(6):631–47. doi: 10.1007/s10654-025-01208-x (PMC12263740; doi:10.1007/s10654-025-01208-x)
Supplement: Supplementary file 1 — Supplementary file1 (DOCX 692 kb) [file 10654_2025_1208_MOESM1_ESM.docx]

Supplementary Figures

Non-linear mendelian randomization: evaluation of effect modification in the residual and doubly-ranked methods with simulated and empirical examples.

Fergus W Hamilton^1,2^, David A Hughes^3^, Tianyuan Lu^4-7^, Apostolos Gkatzionis^1^, Wes Spiller^1^, Kate Tilling^1^, Fernando Pires Hartwig* ^1,8^ George Davey Smith* ^1^

1. MRC Integrative Epidemiology Unit, University of Bristol, Bristol, UK
2. Infection Science, North Bristol NHS Trust, Bristol, UK
3. Pennington Biomedical Research Center, Baton Rouge, LA, USA
4. Lady Davis Institute for Medical Research, Montreal, QC, Canada
5. Department of Statistical Sciences, University of Toronto, Toronto, Canada
6. Department of Population Health Sciences, University of Wisconsin-Madison, Madison, WI, USA
7. Department of Biostatistics and Medical Informatics, University of Wisconsin-Madison, Madison, WI, USA
8. Postgraduate Program in Epidemiology, Federal University of Pelotas, Pelotas, Brazil

*Contributed equally

List of Supplementary Figures:

[Figure 1: A schematic describing the doubly-ranked method. Adapted from^1^. Firstly, participants are ranked by their level of the IV (step 1) into pre-strata. Subseqeuently, within pre-strata, participants are ranked by their level of the exposure (step 2) to generate final strata (step 3). In the figure, a single participant in orange is followed. A more detail exposition is given in the paper introducing the method^2^ 5](#_Toc177988036)

[Figure 2 Simulation results of a linear exposure-outcome association X = 0.3G + 0.3U – 0.02GU + e_x_ and Y = 0.3U + e_Y_. Boxplots represent the estimates from each replicate. In total 100 replicates were performed, with each sample containing 100,000 people. Plot A shows IV-exposure estimates across 10 strata; Plot B shows IV-outcome estimates across 10 strata; Plot C shows MR estimates across 10 strata. The FDR is the proportion of replications that meet a nominal significance for heterogeneity using Cochran’s Q for estimates across strata. The MSE represents the mean squared error in each strata. Hinges represent the 25th and 75th centile, while whiskers represent the point closest to 1.5x the IQR below and above the hinge. Points outside that are plotted individually. 5](#_Toc177988037)

[Figure 3 Simulation results of a linear exposure-outcome association X = 0.3G + 0.3U + e_x_ and Y = 0.3U + e_Y_, Boxplots represent the estimates from each replicate. In total 100 replicates were performed, with each sample containing 100,000 people. Plot A shows IV-exposure estimates across 10 strata; Plot B shows IV-outcome estimates across 10 strata; Plot C shows MR estimates across 10 strata. The FDR is the proportion of replications that meet a nominal significance for heterogeneity using Cochran’s Q for estimates across strata. The MSE represents the mean squared error in each strata. Hinges represent the 25th and 75th centile, while whiskers represent the point closest to 1.5x the IQR below and above the hinge. Points outside that are plotted individually. 7](#_Toc177988038)

[Figure 4 Simulation results of a linear exposure-outcome association X = 0.3G + 0.3U + 0.02GU + e_x_ and Y = 0.3U + e_Y_. Boxplots represent the estimates from each replicate. In total 100 replicates were performed, with each sample containing 100,000 people. Plot A shows IV-exposure estimates across 10 strata; Plot B shows IV-outcome estimates across 10 strata; Plot C shows MR estimates across 10 strata. The FDR is the proportion of replications that meet a nominal significance for heterogeneity using Cochran’s Q for estimates across strata. The MSE represents the mean squared error in each strata. Hinges represent the 25th and 75th centile, while whiskers represent the point closest to 1.5x the IQR below and above the hinge. Points outside that are plotted individually. 8](#_Toc177988039)

[Figure 5 Simulation results of a linear exposure-outcome association X = 0.3G + 0.3U + 0.1GU + e_x_ and Y = 0.3U + e_Y_. Boxplots represent the estimates from each replicate. In total 100 replicates were performed, with each sample containing 100,000 people. Plot A shows IV-exposure estimates across 10 strata; Plot B shows IV-outcome estimates across 10 strata; Plot C shows MR estimates across 10 strata. The FDR is the proportion of replications that meet a nominal significance for heterogeneity using Cochran’s Q for estimates across strata. The MSE represents the mean squared error in each strata. Hinges represent the 25th and 75th centile, while whiskers represent the point closest to 1.5x the IQR below and above the hinge. Points outside that are plotted individually. 11](#_Toc177988040)

[Figure 6 Simulation results of a linear exposure-outcome association X = 0.3G + 0.3U - 0.1GU + e_x_ and Y = e_Y_. Boxplots represent the estimates from each replicate. In total 100 replicates were performed, with each sample containing 100,000 people. Plot A shows IV-exposure estimates across 10 strata; Plot B shows IV-outcome estimates across 10 strata; Plot C shows MR estimates across 10 strata. The FDR is the proportion of replications that meet a nominal significance for heterogeneity using Cochran’s Q for estimates across strata. The MSE represents the mean squared error in each strata. Hinges represent the 25th and 75th centile, while whiskers represent the point closest to 1.5x the IQR below and above the hinge. Points outside that are plotted individually. 13](#_Toc177988041)

[Figure 7 Simulation results of a linear exposure-outcome association X = 0.3G + 0.3U - 0.1GU + e_x_ and Y = e_Y_ , where the MR analyses were partially adjusted for u, by adjusting for a variable that had a correlation of 0.8 with u. Boxplots represent the estimates from each replicate. In total 100 replicates were performed, with each sample containing 100,000 people. Plot A shows IV-exposure estimates across 10 strata; Plot B shows IV-outcome estimates across 10 strata; Plot C shows MR estimates across 10 strata. The FDR is the proportion of replications that meet a nominal significance for heterogeneity using Cochran’s Q for estimates across strata. The MSE represents the mean squared error in each strata. Hinges represent the 25th and 75th centile, while whiskers represent the point closest to 1.5x the IQR below and above the hinge. Points outside that are plotted individually. 15](#_Toc177988042)

[Figure 8 Simulation results of a linear exposure-outcome association X = 0.3G + -0.3U + -0.1GU + 0.3V – 0.1GU + e_x_ and Y = 0.3V + e_Y_ . Boxplots represent the estimates from each replicate. In total 100 replicates were performed, with each sample containing 100,000 people. Plot A shows IV-exposure estimates across 10 strata; Plot B shows IV-outcome estimates across 10 strata; Plot C shows MR estimates across 10 strata. The FDR is the proportion of replications that meet a nominal significance for heterogeneity using Cochran’s Q for estimates across strata. The MSE represents the mean squared error in each strata. Hinges represent the 25th and 75th centile, while whiskers represent the point closest to 1.5x the IQR below and above the hinge. Points outside that are plotted individually. 17](#_Toc177988043)

[Figure 9 Simulation results of a linear exposure-outcome association X = 0.3G + e_x_, but where the variance of e_x_ is related to the level of the IV and Y = e_Y_. Boxplots represent the estimates from each replicate. In total 100 replicates were performed, with each sample containing 100,000 people. Plot A shows IV-exposure estimates across 10 strata; Plot B shows IV-outcome estimates across 10 strata; Plot C shows MR estimates across 10 strata. The FDR is the proportion of replications that meet a nominal significance for heterogeneity using Cochran’s Q for estimates across strata. The MSE represents the mean squared error in each strata. Hinges represent the 25th and 75^th^ centile, while whiskers represent the point closest to 1.5x the IQR below and above the hinge. Points outside that are plotted individually. **Error! Bookmark not defined.**](#_Toc177988044)

[Figure 10: Replication of simulation C in the PLOS Genetics paper with a null outcome. Each facet represents a different number of participants included in each simulation. The Y-axis represents MR betas and interquartile ranges, while the X-axis represents doubly-ranked strata .Each simulation ran 1,000 times, as per the original publication. 20](#_Toc177988045)

[Figure 11: Effect of sickness scores on mortality in UK Biobank. Derivation of these scores described in the methods. 21](#_Toc177988046)

[Figure 12 MR estimates from the confounded empirical analyses. Estimates are from the doubly-ranked method. Stratum specific estimates are on the Y-axis, with the overall effect in the first column, then individual strata specific estimates in each subsequent row. 21](#_Toc177988047)

Figure 1: A schematic describing the doubly-ranked method. Adapted from^1^. Firstly, participants are ranked by their level of the IV (step 1) into pre-strata. Subseqeuently, within pre-strata, participants are ranked by their level of the exposure (step 2) to generate final strata (step 3). In the figure, a single participant in orange is followed. A more detail exposition is given in the paper introducing the method^2^


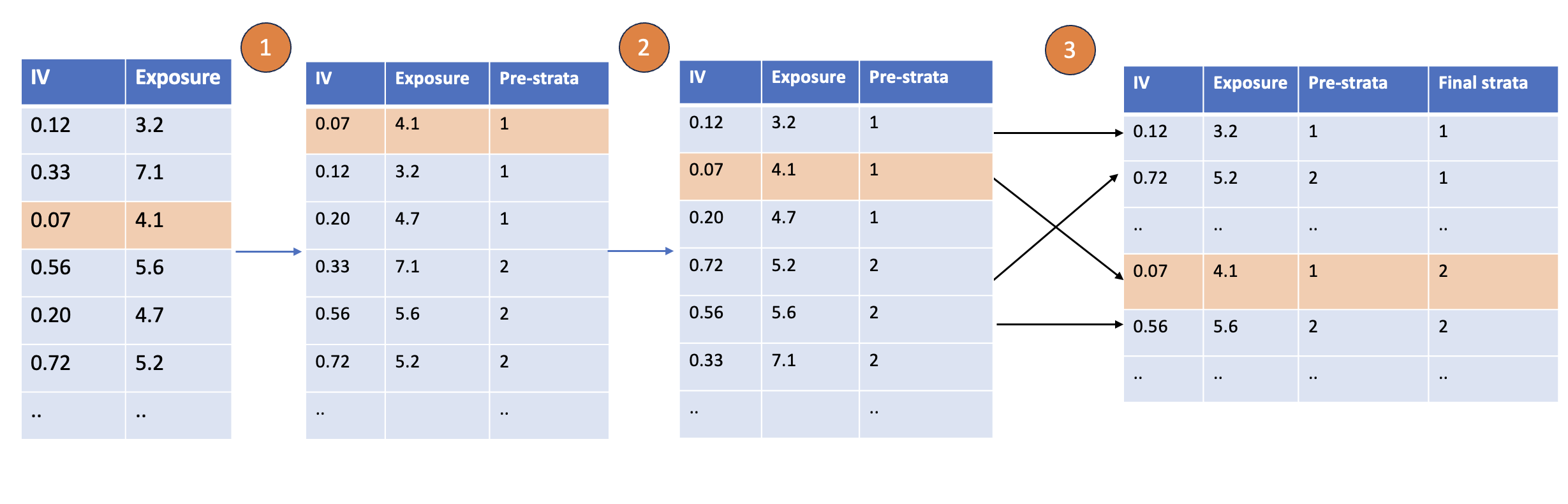


Figure 2 Simulation results of a linear exposure-outcome association X = 0.3G + 0.3U – 0.02GU + e_x_ and Y = 0.3U + e_Y_. Boxplots represent the estimates from each replicate. In total 100 replicates were performed, with each sample containing 100,000 people. Plot A shows IV-exposure estimates across 10 strata; Plot B shows IV-outcome estimates across 10 strata; Plot C shows MR estimates across 10 strata. The FDR is the proportion of replications that meet a nominal significance for heterogeneity using Cochran’s Q for estimates across strata. The MSE represents the mean squared error in each strata. Hinges represent the 25th and 75th centile, while whiskers represent the point closest to 1.5x the IQR below and above the hinge. Points outside that are plotted individually.

Figure 3 Simulation results of a linear exposure-outcome association X = 0.3G + 0.3U + e_x_ and Y = 0.3U + e_Y_, Boxplots represent the estimates from each replicate. In total 100 replicates were performed, with each sample containing 100,000 people. Plot A shows IV-exposure estimates across 10 strata; Plot B shows IV-outcome estimates across 10 strata; Plot C shows MR estimates across 10 strata. The FDR is the proportion of replications that meet a nominal significance for heterogeneity using Cochran’s Q for estimates across strata. The MSE represents the mean squared error in each strata. Hinges represent the 25th and 75th centile, while whiskers represent the point closest to 1.5x the IQR below and above the hinge. Points outside that are plotted individually.

Figure 4 Simulation results of a linear exposure-outcome association X = 0.3G + 0.3U + 0.02GU + e_x_ and Y = 0.3U + e_Y_. Boxplots represent the estimates from each replicate. In total 100 replicates were performed, with each sample containing 100,000 people. Plot A shows IV-exposure estimates across 10 strata; Plot B shows IV-outcome estimates across 10 strata; Plot C shows MR estimates across 10 strata. The FDR is the proportion of replications that meet a nominal significance for heterogeneity using Cochran’s Q for estimates across strata. The MSE represents the mean squared error in each strata. Hinges represent the 25th and 75th centile, while whiskers represent the point closest to 1.5x the IQR below and above the hinge. Points outside that are plotted individually.

Figure 5 Simulation results of a linear exposure-outcome association X = 0.3G + 0.3U + 0.1GU + e_x_ and Y = 0.3U + e_Y_. Boxplots represent the estimates from each replicate. In total 100 replicates were performed, with each sample containing 100,000 people. Plot A shows IV-exposure estimates across 10 strata; Plot B shows IV-outcome estimates across 10 strata; Plot C shows MR estimates across 10 strata. The FDR is the proportion of replications that meet a nominal significance for heterogeneity using Cochran’s Q for estimates across strata. The MSE represents the mean squared error in each strata. Hinges represent the 25th and 75th centile, while whiskers represent the point closest to 1.5x the IQR below and above the hinge. Points outside that are plotted individually.

Figure 6 Simulation results of a linear exposure-outcome association X = 0.3G + 0.3U - 0.1GU + e_x_ and Y = e_Y_. Boxplots represent the estimates from each replicate. In total 100 replicates were performed, with each sample containing 100,000 people. Plot A shows IV-exposure estimates across 10 strata; Plot B shows IV-outcome estimates across 10 strata; Plot C shows MR estimates across 10 strata. The FDR is the proportion of replications that meet a nominal significance for heterogeneity using Cochran’s Q for estimates across strata. The MSE represents the mean squared error in each strata. Hinges represent the 25th and 75th centile, while whiskers represent the point closest to 1.5x the IQR below and above the hinge. Points outside that are plotted individually.

Figure 7 Simulation results of a linear exposure-outcome association X = 0.3G + 0.3U - 0.1GU + e_x_ and Y = e_Y_ , where the MR analyses were partially adjusted for u, by adjusting for a variable that had a correlation of 0.8 with u. Boxplots represent the estimates from each replicate. In total 100 replicates were performed, with each sample containing 100,000 people. Plot A shows IV-exposure estimates across 10 strata; Plot B shows IV-outcome estimates across 10 strata; Plot C shows MR estimates across 10 strata. The FDR is the proportion of replications that meet a nominal significance for heterogeneity using Cochran’s Q for estimates across strata. The MSE represents the mean squared error in each strata. Hinges represent the 25th and 75th centile, while whiskers represent the point closest to 1.5x the IQR below and above the hinge. Points outside that are plotted individually.

Figure 8 Simulation results of a linear exposure-outcome association X = 0.3G + -0.3U + -0.1GU + 0.3V – 0.1GU + e_x_ and Y = 0.3V + e_Y_ . Boxplots represent the estimates from each replicate. In total 100 replicates were performed, with each sample containing 100,000 people. Plot A shows IV-exposure estimates across 10 strata; Plot B shows IV-outcome estimates across 10 strata; Plot C shows MR estimates across 10 strata. The FDR is the proportion of replications that meet a nominal significance for heterogeneity using Cochran’s Q for estimates across strata. The MSE represents the mean squared error in each strata. Hinges represent the 25th and 75th centile, while whiskers represent the point closest to 1.5x the IQR below and above the hinge. Points outside that are plotted individually.

Figure 9: Replication of simulation C in the PLOS Genetics paper with a null outcome. Each facet represents a different number of participants included in each simulation. The Y-axis represents MR betas and interquartile ranges, while the X-axis represents doubly-ranked strata .Each simulation ran 1,000 times, as per the original publication.


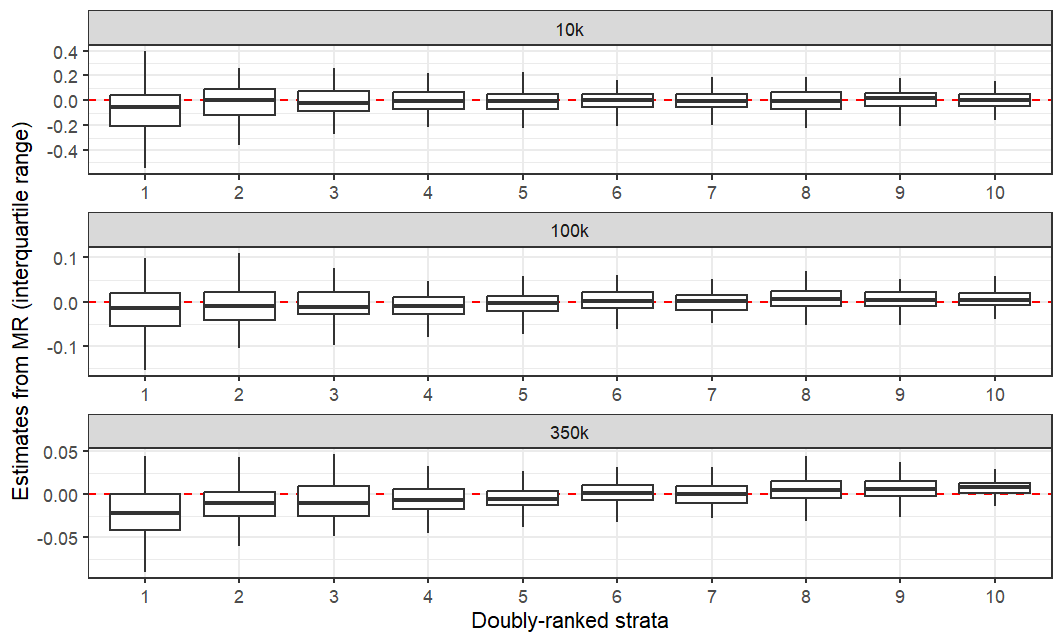


Figure 10: Effect of sickness scores on mortality in UK Biobank. Derivation of these scores described in the methods.

Figure 11 MR estimates from the confounded empirical analyses. Estimates are from the doubly-ranked method. Stratum specific estimates are on the Y-axis, with the overall effect in the first column, then individual strata specific estimates in each subsequent row.


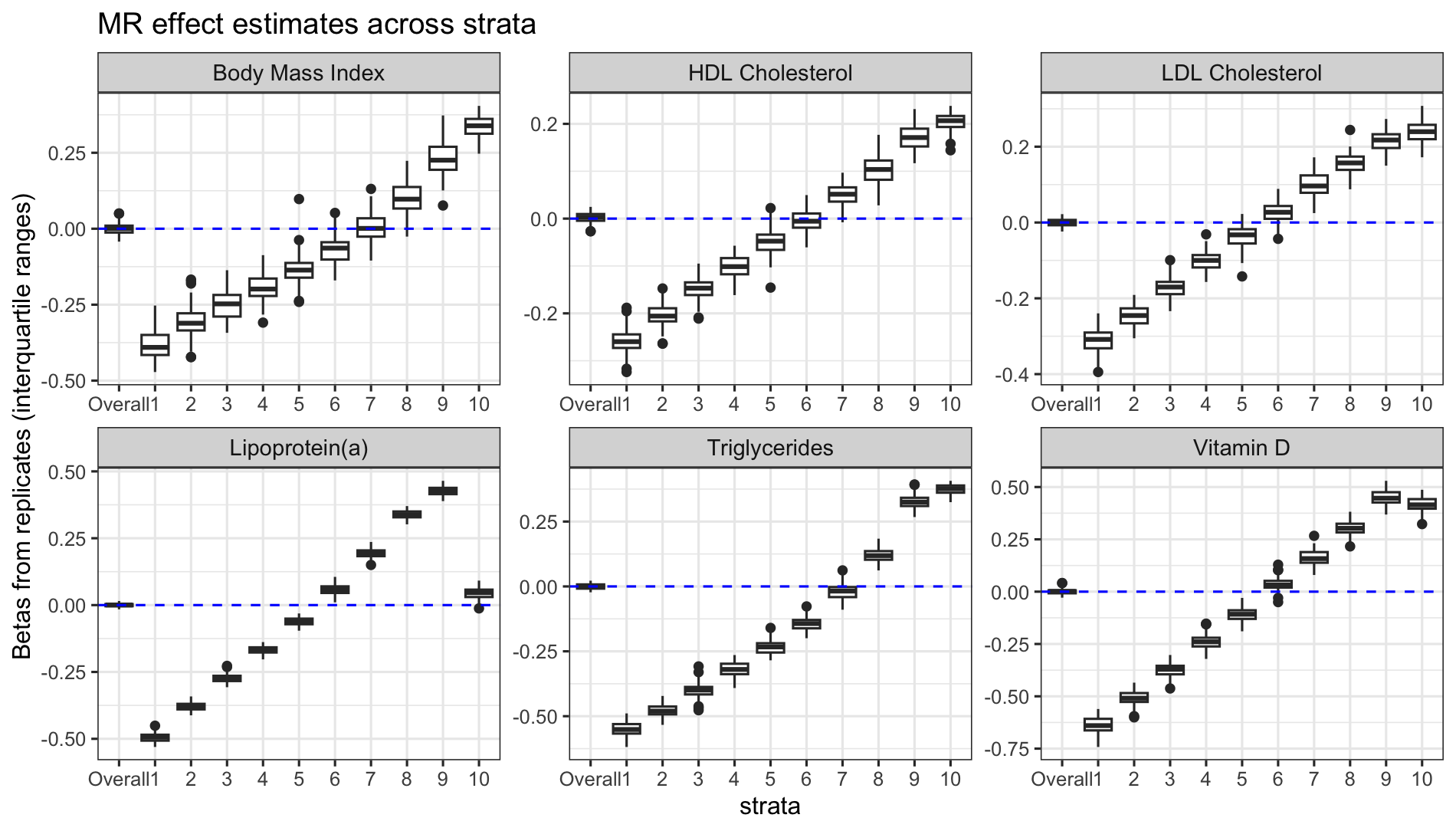


1. Hamilton FW, Hughes DA, Spiller W, Tilling K, Davey Smith G. Non-linear Mendelian randomization: detection of biases using negative controls with a focus on BMI, Vitamin D and LDL cholesterol. Eur J Epidemiol [Internet] 2024;39(5):451–65. Available from: http://dx.doi.org/10.1007/s10654-024-01113-9

2. Tian H, Mason AM, Liu C, Burgess S. Relaxing parametric assumptions for non-linear Mendelian randomization using a doubly-ranked stratification method. PLoS Genet [Internet] 2023 [cited 2023 May 22];19(6):e1010823. Available from: http://dx.doi.org/10.1371/journal.pgen.1010823
