# Supplementary material for: Non-linear Mendelian randomization: evaluation of effect modification in the residual and doubly-ranked methods with simulated and empirical examples
Source: Eur J Epidemiol. 2025 Jun 2;40(6):631–47. doi: 10.1007/s10654-025-01208-x (PMC12263740; doi:10.1007/s10654-025-01208-x)
Supplement: Supplementary file 2 — Supplementary file2 (DOCX 1176 kb) [file 10654_2025_1208_MOESM2_ESM.docx]

Supplementary S1: Individual SNPs and heteroskedasticity

Non-linear mendelian randomization: evaluation of effect modification in the residual and doubly-ranked methods with simulated and empirical examples.

Fergus W Hamilton^1,2^, David A Hughes^3^, Tianyuan Lu^4-7^, Apostolos Gkatzionis^1^, Kate Tilling*^1^, Fernando Pires Hartwig* ^1,8^ George Davey Smith* ^1^

1. MRC Integrative Epidemiology Unit, University of Bristol, Bristol, UK
2. Infection Science, North Bristol NHS Trust, Bristol, UK
3. Pennington Biomedical Research Center, Baton Rouge, LA, USA
4. Lady Davis Institute for Medical Research, Montreal, QC, Canada
5. Department of Statistical Sciences, University of Toronto, Toronto, Canada
6. Department of Population Health Sciences, University of Wisconsin-Madison, Madison, WI, USA
7. Department of Biostatistics and Medical Informatics, University of Wisconsin-Madison, Madison, WI, USA
8. Postgraduate Program in Epidemiology, Federal University of Pelotas, Pelotas, Brazil

*Contributed equally

In this supplement, we explored the effect of heteroscedasticity largely using BMI as an example. We wanted to explore a) whether individual SNPs are randomly distributed across strata, and b) the effect of SNPs in the IV on the IV-exposure effect and MR effects. We first examined whether either the doubly-ranked or residual method truly ensures that the “IV-free” exposure is independent of the SNPs that make up our instrumental variable. If we suspect that effect modification is occurring, we might identify that SNPs in our IV are not randomly distributed across strata.

We extracted all White British participants of UK Biobank and generated doubly-ranked and residual strata for each participant using a PRS generated from 67 SNPs, with weighting of each SNP from data external to UK Biobank. The 67 SNPs were the same SNPs as used to generate our PRS in our main analyses. We then calculated the effect allele frequency for each SNP across strata, and performed a Chi-Square test to test whether these were uncorrelated with strata membership.

In both the doubly-ranked and residual stratification approaches, we see that the SNPs that make up our instrumental variable are not all distributed randomly across strata (Figure 2). In general, evidence of non-random distribution of SNPs across strata was much stronger in the residual method than the doubly-ranked, but there was still clear evidence that SNPs were unevenly distributed across strata in the doubly-ranked method, with clear inflation of quantile-quantile (QQ) plots (Figure 1) and with 15 of the 68 SNPs meeting a nominal significance threshold (p < 0.05).

Figure 2 Plot A shows the P-value from the Chi-Sq test for the residual method (x-axis) and the ranked method (y-axis) of each SNP across strata of BMI. Nominal significance thresholds are drawn in blue, and the red line represents x=y. Note, this plot is truncated to exlude one outlier SNP (rs1558902), which has an extremely low p-value in the residual method. Plot B shows the allele frequency of this SNP(rs1558902) across strata. Plot C and D shows the allele frequency of two other SNPs that had strong evidence of different allele frequencies across strata in both methods.

Figure 1: QQ-plots of the p-values of the association between SNPs in the BMI IV and strata of BMI definied by the residual method and the ranked method.

One SNP in particular (rs1558902, Figure 2) had extreme evidence of differing allele frequencies across strata in the residual method (p = 3.5x 10^-80^) with much weaker (but some) evidence of difference in the doubly-ranked method (p = 0.004). This SNP – within *FTO* – has the largest effect on BMI of all included SNPs. It is also worth noting that the association between this SNP and strata membership is much weaker when testing a linear model: i.e, when regressing the numeric value of strata (e.g. 1,2…10) onto rs1558902, results for the doubly-ranked method were null (beta 0.002, 95% CI -0.011; 0.014, p = 0.79), and much weaker for the residual method (beta -0.02; 95% CI -0.034; -0.01, p = 0.001). That is, carriers of the BMI increasing allele at *FTO* are more common in extreme strata, but there is much weaker evidence that they are more common in lower strata than higher strata.

We also noted that the P-value for Glejser’s test – a test of heteroskedasticity - of the effect of this SNP on BMI at rs1558902 was 3 x 10^-100^, supporting our simulation findings that heteroskedasticity might influence the assortment of individual SNPs into strata. Taking this further, we compared the (logged) P-value for Glejsers test against the (logged) P-value for the Chi-Sq test comparing allele frequencies across strata. In the residual method we found very high correlation (Pearson’s correlation of logged p-values 0.95, p 2 x 10^-35^), although there was little evidence of a correlation for the doubly-ranked method (r = 0.15, p = 0.16). This data suggests that for the residual method, there is a strong relationship between the heteroscedasticity of individual SNP exposure relationship and whether these SNPs are randomly distributed across strata.

We wondered whether there might be other genetic associations with being in extreme strata outside the IV. We therefore performed a GWAS of a) raw BMI, residual strata order, ranked strata order, and a case-control study of being in a extreme strata (1 or 10) versus being in a middle strata for both methods. Analysis was performed in the European subset of UK Biobank, with further details in the methods. In the residual method we identified a number of genetic associations across the genome for being in extreme strata that met a nominal GWAS significance threshold (including rs1558902 at *FTO*, Manhattan plots in Figure 3).

Figure 3: Manhattan plots of GWAS perfomed in this study. A: Raw BMI, B, GWAS of residual strata, C: GWAS of ranked strata, D: GWAS of extreme residual strata, and E: GWAS of extreme ranked strata.


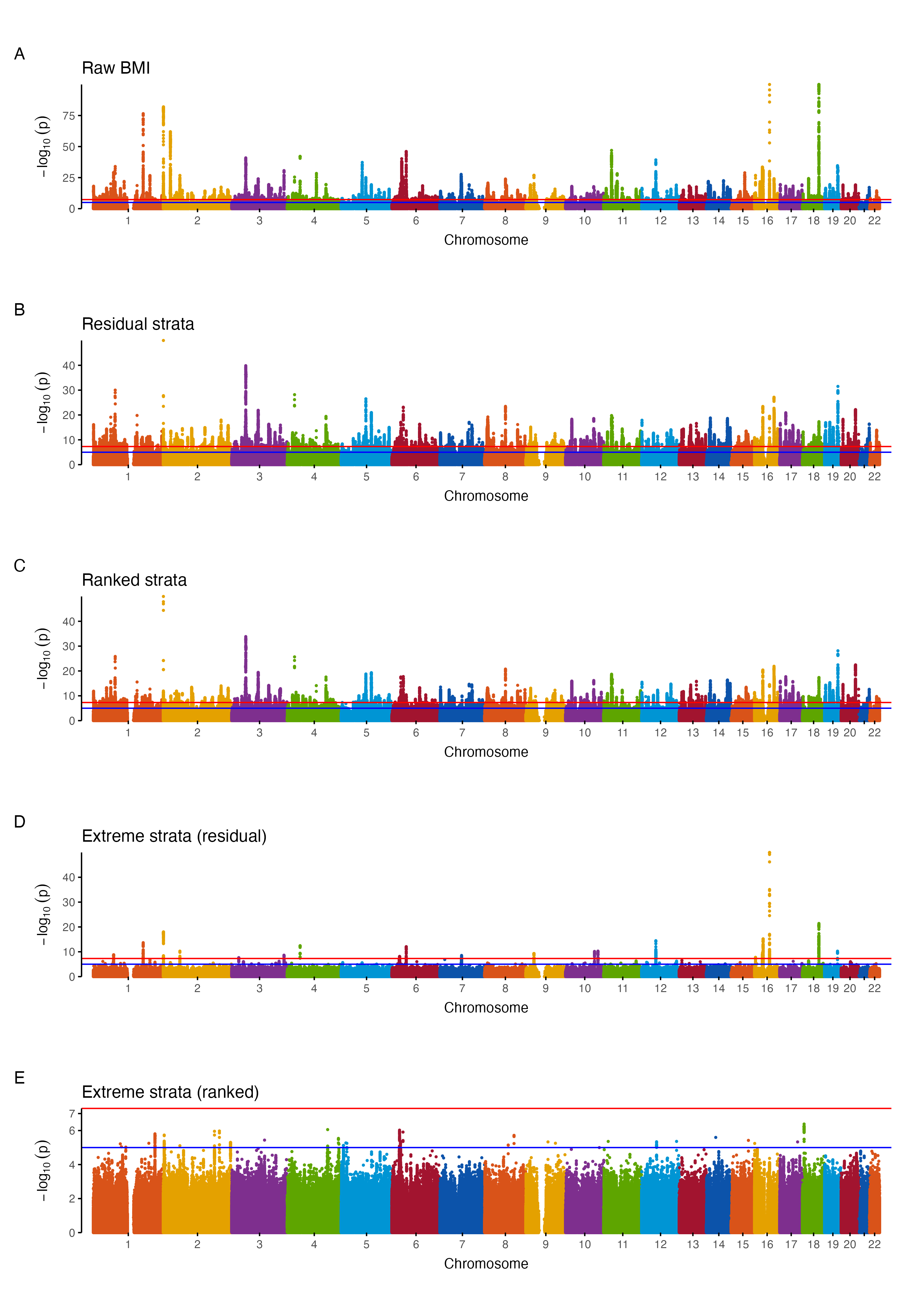


For the doubly-ranked method, we did not identify any associations that met genome-wide significance, but at the *FTO* locus, the lowest p value was 9 x 10^-5^, suggesting that carriers of certain *FTO* genotypes which are not included in our IV are more likely to be in extreme strata of the doubly-ranked method also.

The above analyses raised concern around whether MR estimates generated by the NLMR methods would be biased in the analysis of BMI as an exposure and whether identification and removal of SNPs that exhibited heteroscedasticity could potentially reduce bias. These SNPs may be less sensitive to interaction with other variables or may have less inherent effect on the variance of BMI.

To test this, we tried to limit heteroskedasticity in our IV. For all participants with a measured BMI and genetic data we tested each SNP in our IV in turn against the exposure, to identify the degree of heteroskedasticity. Remarkably, 63/68 SNPs met a nominal threshold for heteroscedasticity (Glejser’s P <0.05). We then generated three IV’s from the SNPs: one including all SNPs, one including SNPs that had a Glejser P > 1 x 10^-5^, and one that only included SNPs that had Glejser P > 0.05. We then summed the alleles scores of included SNPs to generate our IVs. These IV’s included 68, 25 and 5 SNPs respectively, and had F-statistics of 6,867, 1,652, and 154 respectively. Despite including SNPs that had low heteroskedasticity, the p-values for the Glejser test for each IV representing the summed allelic scores were low (<1 x 10^-300^, 6 x 10^-65^, 0.007, respectively) We then performed an analysis using a simulated, confounded exposrure outcome as described in the manuscript, where we added confounding between BMI and a simulated exposure to generate a situation where bias could only be generated by the genotype-exposure effect. We fixed bux = -1, and buy at 1. As expected, linear MR estimates were null (Figure 4) using all three IV’s, although IV-exposure estimates varied across strata depending on the IV, with much more compelling evidence of increasing genetic effect with the IV containing all SNPs. Strata-specific MR estimates regardless of the PRS chosen, but there appeared to be less evidence of strata-specific bias in MR estimates when limiting the PRS to those SNPs that had lower heteroskedasticity.

We then performed a similar approach for another commonly used exposure - high density lipoprotein (HDL) and found similar results (Figure 5 )with a decrease in the ‘increasing genetic effect’ when limiting our IVs to SNPs that do not have heteroskedasticity, and strata specific MR estimates that were closer to the null, although the PRS with fewer SNPs were, by nature, weaker and therefore estimates were necessarily less precise. For example, in the IV containing all SNPs the estimated gene exposure effect increases broadly linearly from 0.5 in strata 1 to 1.5 in strata 10 (3-fold increase), whereas in the IV that contains only SNPs with limited heteroscedasticity the gene-exposure effect is similar the middle 8 strata, and has a reduced difference of 0.8 in the bottom strata to 1.4 in the top strata (~1.5 fold increase).

We recognize that selectively identifying SNPs by lack of heteroskedasticity may generate bias and will definitely reduce power do not here recommend it as a method, but simply note that the size of the ‘increasing genetic effect’ reported for BMI by the authors of the doubly-ranked method^43^ appears to depend on whether the IV includes SNPs that are heteroskedastic, which in our simulations above, is likely to generate IV-exposure estimates that are non-constant across strata, while also apparently reducing some bias in MR estimates in some settings. Further investigations of this approach are warranted.

We attempted to perform the same analysis for Vitamin D but all SNPs in our PRS except rs4081429 had evidence for heteroskedasticity and so we were unable to generate a reduced PRS. Consistent with this heteroskedasticity, the distribution of SNPs across each strata was not random, which we show in Figure 6.

Figure 4 Simulation results of a BMI onto a random, normally distributed outcome. Plot A shows IV-exposure estimates across 10 strata; Plot B shows IV-outcome estimates across 10 strata; Plot C shows MR estimates across 10 strata. The red blocks represent the PRS limited to SNPs with limited heteroskedascity, the green blocks the SNPs with some heteroskedascticity, and the blue blocks represent the IV including all SNPs.

Figure 5: Simulations of a confounded HDL outcome, where confounding has been added to the exposure and outcome. Plot A shows IV-exposure estimates across 10 strata; Plot B shows IV-outcome estimates across 10 strata; Plot C shows MR estimates across 10 strata. The red blocks represent the PRS limited to SNPs with some heteroskedascity, the green blocks the SNPs with the least heteroskedasticity, and the blue blocks represent the IV including all SNPs.

Figure 6: Plot A shows the P-value from the Chi-Sq test for the residual method (x-axis) and the ranked method (y-axis for each SNP across strata of Vitamin D. Nominal significance thresholds are drawn in blue, and the red line represents x=y. Plots, B, C and D show the allele frequency of two other SNPs that had strong evidence of different allele frequencies across strata in both methods.
