# Supplementary material for: Non-linear Mendelian randomization: evaluation of effect modification in the residual and doubly-ranked methods with simulated and empirical examples
Source: Eur J Epidemiol. 2025 Jun 2;40(6):631–47. doi: 10.1007/s10654-025-01208-x (PMC12263740; doi:10.1007/s10654-025-01208-x)
Supplement: Supplementary file 4 — Supplementary file4 (DOCX 24 kb) [file 10654_2025_1208_MOESM4_ESM.docx]

Supplementary Notes

Non-linear mendelian randomization: evaluation of effect modification in the residual and doubly-ranked methods with simulated and empirical examples.

Fergus W Hamilton^1,2^, David A Hughes^3^, Tianyuan Lu^4-7^, Apostolos Gkatzionis^1^, Kate Tilling*^1^, Fernando Pires Hartwig* ^1,8^ George Davey Smith* ^1^

1. MRC Integrative Epidemiology Unit, University of Bristol, Bristol, UK
2. Infection Science, North Bristol NHS Trust, Bristol, UK
3. Pennington Biomedical Research Center, Baton Rouge, LA, USA
4. Lady Davis Institute for Medical Research, Montreal, QC, Canada
5. Department of Statistical Sciences, University of Toronto, Toronto, Canada
6. Department of Population Health Sciences, University of Wisconsin-Madison, Madison, WI, USA
7. Department of Biostatistics and Medical Informatics, University of Wisconsin-Madison, Madison, WI, USA
8. Postgraduate Program in Epidemiology, Federal University of Pelotas, Pelotas, Brazil

*Contributed equally

**Supplementary Note 1: Proof that non-linear genotype-exposure relationships do not violate the rank-preserving assumption.**

**Proposition:** Let $X$ be the exposure, $G$ the genetic IV and $U$ other causes of $X$. So $X=f_{X}\left( G,U \right)$, where $f_{X}$ is the function describing how $G$ and $U$ determine $X$. Assume the dose-response curve describing the additive effect of $G$ on $X$ is the same for everyone in the studied population. Then, the rank-preserving assumption holds.

**Proof:** The rank-preserving assumption can be expressed as follows: $f_{X}\left( G=g,U=u \right)>f_{X}\left( G=g,U=u^{'} \right)\Leftrightarrow f_{X}\left( G=g^{'},U=u \right)>f_{X}\left( G=g^{'},U=u^{'} \right)$, for all values of $g$, $g^{'}$, $u$ and $u^{'}$. The statement that the dose-response curve describing the additive effect of $G$ on $X$ is the same for everyone in the studied population can be expressed as follows: $f_{X}\left( G=g,U=u \right)-f_{X}\left( G=g^{'},U=u \right)=f_{X}\left( G=g,U=u^{'} \right)-f_{X}\left( G=g^{'},U=u^{'} \right)$ for all values of $g$, $g^{'}$, $u$ and $u^{'}$. Since the additive effect of $G$ on $X$ does not depend on $U$, it can be described by a function solely in terms of $G$, which we denote $k\left( G \right)$ – i.e., $k\left( G=g \right)-k\left( G=g^{'} \right)=f_{X}\left( G=g,U=u \right)-f_{X}\left( G=g^{'},U=u \right)$ for all values of $g$, $g^{'}$ and $u$. Equivalently, the additive effect of $U$ on $X$ does not depend on $G$. Thus, it can be described by a function solely in terms of $U$, which we denote $j\left( U \right)$ – i.e., $j\left( U=u \right)-j\left( U=u^{'} \right)=f_{X}\left( G=g,U=u \right)-f_{X}\left( G=g,U=u^{'} \right)$ for all values of $u$, $u^{'}$ and $g$. Therefore:

$$f_{X}\left( G=g,U=u \right)-f_{X}\left( G=g^{'},U=u^{'} \right)=$$

$$f_{X}\left( G=g,U=u \right)-f_{X}\left( G=g^{'},U=u \right)+f_{X}\left( G=g^{'},U=u \right)-f_{X}\left( G=g^{'},U=u^{'} \right)=$$

$$f_{X}\left( G=g,U=u \right)-f_{X}\left( G=g^{'},U=u \right)+f_{X}\left( G=g^{'},U=u \right)-f_{X}\left( G=g^{'},U=u^{'} \right)=$$

$$k\left( G=g \right)-k\left( G=g^{'} \right)+ j\left( U=u \right)-j\left( U=u^{'} \right)\Rightarrow$$

$$f_{X}\left( G=g,U=u \right)=f_{X}\left( G=g^{'},U=u^{'} \right)+k\left( G=g \right)-k\left( G=g^{'} \right)+ j\left( U=u \right)-j\left( U=u^{'} \right)$$

$$f_{X}\left( G=g,U=u \right)=\left[ f_{X}\left( G=g^{'},U=u^{'} \right)-k\left( G=g^{'} \right)-j\left( U=u^{'} \right)+k\left( G=g \right) \right]+ j\left( U=u \right)$$

$f_{X}\left( G=g,U=u \right)=h\left( G=g \right)+ j\left( U=u \right)$,

where $h\left( G=g \right)=f_{X}\left( G=g^{'},U=u^{'} \right)-k\left( G=g^{'} \right)-j\left( U=u^{'} \right)+k\left( G=g \right)$.

The above shows that, if the additive causal dose-response curve is constant, $f_{X}\left( G,U \right)= h\left( G \right)+j\left( U \right)$. Therefore:

$$f_{X}\left( G=g,U=u \right)>f_{X}\left( G=g,U=u^{'} \right)\Leftrightarrow$$

$$h\left( g \right)+j\left( u \right)>h\left( g \right)+j\left( u^{'} \right)\Leftrightarrow$$

$$j\left( u \right)>j\left( u^{'} \right)\Leftrightarrow$$

$h\left( g^{'} \right)+j\left( u \right)>h\left( g^{'} \right)+j\left( u^{'} \right)\Leftrightarrow f_{X}\left( G=g^{'},U=u \right)>f_{X}\left( G=g^{'},U=u^{'} \right)$. Then, the rank-preserving assumption holds.

**Supplementary Note 2: Simulated exposures and simulated outcomes in UK Biobank.**

Figure 10 displays the Directed Acyclic Graph (DAG) that corresponds to the analysis of simulated exposures and simulated outcomes. In the DAG, G represents the observed genetic instrumental variable (IV), specifically the exposure allele score in the UKB. X denotes the observed exposure, which is measured in the UKB. X^S^, U^S^, and Y^S^ represent the simulated exposure, confounder, and outcome, respectively. The dashed line connecting G and X signifies that G and X are associated, but through an unknown causal mechanism.

We now use this DAG to demonstrate that, in this simulation, G is a valid IV (i.e., it satisfies the three core IV assumptions of relevance, independence and exclusion restriction) for the effect of X^S^ on Y^S^. Relevance is clearly satisfied since G and X are d-connected, X and X^S^ are d-connected, and X cannot be a collider between G and X^S^ because X causes X^S^. Independence and exclusion restriction hold because G and Y^S^ are d-separated when X^S^ does not cause Y^S^ (as in Figure 11). To see this, note that X^S^ is influenced by both X and U^S^, making it a collider in the only path between G and Y^S^. Therefore, the only path between G and Y^S^ is blocked, implying that G and Y^S^ are d-separated and, as a result, independent.^1^

This result proves that, in this analysis, any selection bias (or any other factor) affecting the relationship between G and X in the UKB cannot result in G being an invalid IV for the effect of X^S^ on Y^S^ (indeed, conventional MR analyses applied to these simulations were null). Since the effect X^S^ on Y^S^ is null for everyone in this simulation, causal effect homogeneity holds. Therefore, any bias we are observing when applying non-linear MR methods must be driven by violations of IV4 assumptions (since the core IV assumptions hold) required by these methods that related to the G-X association (since the X-X^S^ and X^S^-Y effects are homogeneous): namely, the constant genetic effect assumption for the residual method and the rank-preserving assumption for the doubly-ranked method. It should be noted that this result does not preclude the possibility of such IV4 assumptions being violated because of selection bias. For instance, if G causally influences selection, then G could become associated with another cause of selection that affects the variance of X, potentially leading to heteroscedasticity in X relative to G.

Importantly, while the selection process might elucidate why heteroscedasticity exists, it would not serve as an alternative explanation for the NLMR bias itself. In essence, the selection-induced relationship between G and the variance of X could create the conditions for bias in NLMR, but the direct cause of this bias would remain the resulting heteroscedasticity.

1. Hernan MA, Robins JM. Causal Inference [Internet]. CRC Press; 2023. Available from: https://play.google.com/store/books/details?id=_KnHIAAACAAJ
